# Evolution of Charge and Orbital Ordering, and Cation Vacancy Ordering During Electrochemical Desodiation of Na_*x*_NiO_2_

**DOI:** 10.1021/jacs.6c03074

**Published:** 2026-05-20

**Authors:** James M. A. Steele, Joshua D. Bocarsly, Liam A. V. Nagle-Cocco, George S. Phillips, Farheen N. Sayed, Giulio I. Lampronti, Fabio Orlandi, Pascal Manuel, Iuliia Mikulska, Clare P. Grey, Siân E. Dutton

**Affiliations:** † Yusuf Hamied Department of Chemistry, 2152 University of Cambridge, Cambridge CB2 1EW, U.K.; ‡ Cavendish Laboratory, University of Cambridge, JJ Thomson Avenue, Cambridge CB3 0US, U.K.; § Department of Chemistry and Texas Center for Superconductivity, 14743 University of Houston, Houston, Texas 77004, United States; ∥ Department of Materials Science and Metallurgy, University of Cambridge, Cambridge CB3 0FS, U.K.; ⊥ ISIS Neutron and Muon Source, Rutherford Appleton Laboratory, STFC, UKRI, Harwell Science and Innovation Campus, Didcot OX11 0QX, U.K.; # Diamond Light Source, Harwell Science and Innovation Campus, Didcot OX11 0DE, U.K.

## Abstract

NaNiO_2_ is a promising cathode material for sodium-ion batteries due to its high theoretical capacity of 235.8 mAh.g^–1^. However, as with many Na-ion cathode materials, a series of poorly understood phase transitions occur on electrochemical cycling, inducing volume mismatch-based stress/strain, resulting in particle cracking, electrochemically disconnected particles and, therefore, irreversible capacity loss. This behavior is one key obstacle to developing long-lasting, high-performance Na-ion batteries. Although the series of phases that form as Na_*x*_NiO_2_ is electrochemically cycled have been previously identified, their structures remained unsolved, limiting our ability to understand and control the phase transition behavior. Here, we report structural solutions based on Rietveld refinement against high-resolution synchrotron x-ray diffraction (SXRD) and neutron powder diffraction (NPD) for the phases obtained on desodiation: P″3-Na_1/2_NiO_2_, O″3-Na_2/5_NiO_2_, and O‴3-Na_1/3_NiO_2_. Each phase contains a unique Na^+^/vacancy ordering, minimizing intralayer electrostatic repulsions between Na^+^ ions, and Ni^*x*+^-charge ordering decreasing interlayer repulsions through the location of lower valence Ni^*x*+^ nearer to vacancies. Using these structures, we conduct sequential Rietveld refinement against *operando* SXRD data, which supports prior identification of a transient P‴3-Na_1/2<*x*<2/3_NiO_2_ phase, not isolable *ex situ*. *Operando* data also identify the presence of a solid-solution phase O″3δ-Na_1/3<*x*<2/5_NiO_2_ and second-order behavior of the O″3-Na_2/5_NiO_2_ → O‴3-Na_1/3_NiO_2_ phase transition at the top of charge. This work provides unprecedented insight into structural evolution during electrochemical cycling in Ni-rich Na cathodes (and likely Li analogues), paving the way toward rational doping regimes designed to disrupt degradation-inducing phase transitions, increasing capacity and cycle lifetime, thus improving the performance of Co-free Na and Li cathodes.

## Graphical abstract

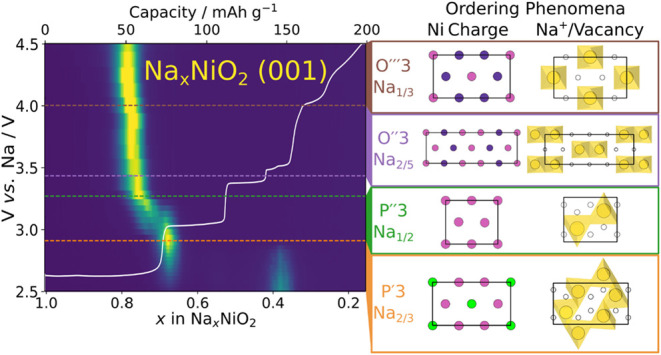

## Introduction

Na-ion batteries can, in principle, provide significant cost-saving and sustainability improvements over Li-ion batteries, owing primarily to the lower cost, greater abundance, and more uniform geographical distribution of sodium.^1^ Unfortunately, many Na-ion batteries are currently hindered by shorter lifetimes and poor cyclability due to the numerous biphasic structural transitions that Na-ion cathode materials undergo during cycling. This degradation is exacerbated by the increased size of Na^+^ (vs. Li^+^ *)*, resulting in greater stress/strain-induced particle cracking, leading to disconnected active material and forming “dead” regions, causing irreversible capacity loss.^2^ Further, since the greater size and mass of Na^+^ places inherent limitations on the volumetric/specific capacities of Na vs. analogous Li cathodes, optimization of performance, stability, and reversibility is crucial if Na-ion batteries are to realize their full potential in applications where low-cost and enhanced sustainability are key figures of merit.

High-Ni-content cathodes have drawn significant interest in Na-ion and Li-ion cathode research due to the possibility of increasing energy density, while decreasing reliance on more expensive and scarce resources such as cobalt.^3,4^ Within this framework, both NaNiO_2_ and LiNiO_2_ are promising cathode materials, owing to their high theoretical capacities of ∼236 and ∼274 mAh.g^–1^ respectively.^5^ However, nickel layered-transition metal oxides undergo large volume change effects due to the combined effect of alkali-ion extraction and oxidation of the Jahn–Teller (JT) active d^7^ Ni^3+^ to the non-JT active d^6^ Ni^4+^ ion.^6^ To date, it has only been possible to achieve a limited reversible capacity of 120 mAh.g^–1^ in NaNiO_2_, as cycling is restricted to the voltage range 1.25–3.75 V so as to improve capacity retention, though long-term cycling is likely to be hindered by the range of biphasic transitions observed throughout dis/charge.^7^ This is only marginally worse than the reversible capacity of 135 mAh.g^–1^ obtained to date in LiNiO_2_ after 400 cycles.^5^ However, it is noted that through further limiting the voltage window (2.7–4.1 V vs Li/Li^+^) to avoid the H2–H3 transition in LiNiO_2_, it is possible to achieve stable reversible capacities of up to 168 mAh.g^–1^.^8^

In stoichiometric NaNiO_2_, the cooperative JT distortion results in collinear cooperative ordering of NiO_6_ octahedra, manifesting a monoclinic (*C*2/*m*) structure.^9,10^ A complex series of distinct layer-glide transitions have been proposed to occur during electrochemical cycling of NaNiO_2_. The structures formed were described using Delmas’ notation,^11^ which labels each phase as *Xn*, where *X* is O or P for octahedral/prismatic coordination of the Na^+^ ions, and *n* is a number denoting how many *TM*O_2_ slab layers are required to describe the stacking sequence of the unit cell (*i*.*e*. O′3, O = octahedrally coordinated Na^+^, 3 = ABC layer stacking, ′ = monoclinic distortion from ideal O3 structure with rhombohedral *R*3̅*m* symmetry). Additional primes are used to distinguish phases with different cation contents, but with otherwise the same Delmas’ notation. The transitions between these phases control the cycling behavior of NaNiO_2_, yet their structures remain unknown, limiting our ability to understand or control the phase transitions and cycling behavior in Na_*x*_NiO_2_.

To fully elucidate the structures of these phases, accurate modeling of the weak Na^+^/vacancy- and Ni^*x*+^ charge-ordering superstructure peaks are required. The oxide-ion positions also need to be determined to high accuracy, so as to interrogate the distorted/orbitally ordered NiO_6_ environments and provide, with any degree of confidence, assignment of Ni-charge states from bond valence sum (BVS) calculations (exact methodology described in the Supporting Information of our prior report).^12^ In a previous study, de Boisse used Pawley fitting of X-ray diffraction (XRD) data to identify lattice parameters and possible space groups for the P′3-Na_2/3_NiO_2_, P″3-Na_1/2_NiO_2_, O″3-Na_2/5_NiO_2_ phases.^13^ The proposed cells are expanded in the *b*-direction relative to the parent structure and retain a monoclinic distortion. The Supporting Information of Chen et al. reported Rietveld refinement of *in situ* synchrotron X-ray diffraction (SXRD),^14^ providing preliminary structures for P′3-Na_2/3_NiO_2_, P″3-Na_1/2_NiO_2_, and a further desodiated phase, O‴3-Na_1/3_NiO_2_. However, their refinements did not reproduce the observed superstructure peaks well, and the structures generated with their reported atom positions result in unphysical Ni–O and Na–O bond lengths, motivating further studies.

Recently, we developed a robust methodology to facilitate neutron powder diffraction (NPD) and high-resolution *ex situ* SXRD on electrochemically derived phases. We used this approach to determine a complete structural solution for P′3-Na_2/3_NiO_2_, the first desodiated phase formed on charging.^12^ By utilizing the WISH diffractometer at the ISIS Neutron and Muon Source,^15^ with its high signal-to-noise at low *Q*, and exceptional neutron flux, we were able to observe superstructure peaks in the NPD data on these relatively small (∼100 mg) electrochemically derived samples, allowing for accurate determination of the oxygen positions. Correlated Na^+^/vacancy ordering and Ni^*x*+^ charge ordering was found in P′3-Na_2/3_NiO_2_, with ordering being highly dynamic at room temperature.

Here we extend our structural study and perform symmetry-informed combined Rietveld refinements of models against SXRD and NPD data to derive, for the first time, complete atomistic structural solutions of the remaining phases, which were not provided by the prior approaches. Specifically, we report complete atomistic structures for P″3-Na_1/2_NiO_2_, O″3-Na_2/5_NiO_2_, and O‴3-Na_1/3_NiO_2_. The same space group and cell parameters are used for the P″3/O″3 cells as those obtained from Pawley analysis,^13^ while we find a novel cell for the most desodiated phase (O‴3). Each cell has a unit cell expansion in the *b*-lattice direction (with respect to the parent NaNiO_2_ unit cell) of 2 × *b*_O′3_, 5 × *b*_O′3_, and 3 × *b*_O′3_ for Na_*x*_NiO_2_ where x = 1/2, 2/5, and 1/3 respectively. We find discrete, stable ordering of Na^+^/vacancies in the Na layer of each phase; these minimize electrostatic repulsive interactions between Na^+^ ions. Ni-charge/orbital ordering in NiO_6_ octahedra is also found in the O″3 and O‴3 structures. *Operando* SXRD provides evidence of a transient metastable P‴3-Na_1/2<*x*<2/3_NiO_2_ phase between P′3-Na_2/3_NiO_2_ and P″3-Na_1/2_NiO_2_, which is not isolable *ex situ*, as well as evidence of the solid-solution/second-order behavior of the O″3-Na_2/5_NiO_2_ → O‴3-Na_1/3_NiO_2_ phase transition at the top of charge, as had previously been suggested in literature.^6,16^ Our results provide evidence of the contributions to desodiated Na_*x*_NiO_2_ structures from orbital-, charge- and Na^+^/vacancy-ordering providing insight into the structural evolution during electrochemical cycling in Ni-rich Na cathodes, and an analogue for defect-free Li equivalents.

## Experimental Methods

### NaNiO_2_ Synthesis

NaNiO_2_ was synthesized *via* a solid-state route as per our prior report.^12^ All reactant powders (NiO *Alfa Aesar Puratronic 99*.*998%*, Na_2_O_2_ *Sigma 97%*) including 5 wt % excess sodium source to mitigate evaporation of volatile Na during synthesis, were mixed and ground manually using an agate pestle and mortar for 15 min, before being pelletized using a pellet press at approximately 5 MPa, then transferred to an alumina crucible, all within an Ar-filled glovebox. All syntheses were carried out at 700 °C for 10 h (ramp rate = 3 °C min^–1^), cooling ramp rate set to the maximum (10 °C min^–1^). In the absence of active cooling, this resulted in cooling at ambient rate. The entire synthesis was conducted under continuous O_2_ with a flow rate of approximately 30 mL min^–1^. Air/moisture exposure was minimized through allowing the sample to fully cool to room temperature before opening the furnace, followed by rapid transfer of the product from the furnace to an Ar-filled glovebox, though it was not possible to eliminate exposure completely.

Following this, all samples were stored and handled within an Ar-filled glovebox at all times.

### Cell Fabrication, Electrochemical Cycling, and *Ex Situ* and *Operando* Sample Preparation

In order to prepare large sample quantities of the desodiated phases suitable for NPD, we used 1″ diameter Swagelok cells (SI of Steele *et. al.*).^12^ The synthesized active cathode material (NaNiO_2_) and conductive carbon (Super P) were mixed in a 70:30 ratio for ∼15 min using an agate pestle and mortar, then the resultant powder was added directly to the Swagelok stainless steel current collector. The cells were assembled in the following order, starting from the stainless steel plunger, stainless steel current collector, cathode/carbon mixture, 2 × 1″ fiber glass separator, 400 μL electrolyte (1 M NaPF_6_ in propylene carbonate [PC], produced as required to minimize degradation), 15/16″ Na metal anode, stainless steel current collector, a rigid spring, and stainless steel plunger. The body of the cell was wrapped with Kapton film internally to prevent short-circuiting/degradation. Na metal anodes were produced at the time of use as follows: since the Na-metal is stored in mineral oil, the oil was first washed off in a glass vial using heptane, before the metal was rolled to a suitable thickness, and punched manually with a 15/16″ manual punch. *Ex situ* cells were cycled using a Biologic potentiostat/galvanostatic at rates of C/100 as per the methodology reported in our prior work.^12^

*Operando* electrochemical SXRD measurements were performed using a bespoke cell designed in our laboratory (SI Section S-1, Figure S1), designed to keep conditions as similar as possible to that of a standard 2032 coin cell. The cell was assembled with 13 mm cathode films (80:10:10 cathode, carbon, PVDF binder, cast onto Al film using a 300 μm doctor blade in Ar atmosphere), ${\frac{5}{8}}^{\prime \prime }$ glass fiber separator, and 150 μL electrolyte (1 M NaPF_6_ in propylene carbonate [PC]). For more information on casting of NaNiO_2_ slurries, see SI of Steele *et. al*.^12^ In order to improve the signal-to-noise (SNR) of the diffraction experiment, 3 stacked cathode films (peeled from the Al current collector initially cast upon) were used instead of a single electrode, resulting in a total active mass of 24.4 mg. The glass window was glued into the outer steel and PEEK body with epoxy to prevent electrolyte leakage, and pressure was maintained *via* the tightening of 4 outer screws into the PEEK cell body, sealing the cell with a Viton rubber O-ring. *Operando* cells were cycled using a Neware potentiostat at a rate of C/10 at the I11 beamline at Diamond Light Source.^17^

### Ni K-Edge X-ray Absorption Near Edge Structure

X-ray absorption spectroscopy (XAS) measurements were performed on the B18 beamline at Diamond Light Source, U.K.^18,19^ Na_*x*_NiO_2_ samples were mixed homogeneously with a dried, powdered cellulose binder (∼3 mg: ∼30 mg Na_*x*_NiO_2_:Cellulose) and pressed into a ${\frac{1}{4}}^{\prime \prime }$ pellet in an Ar-filled glovebox. Pellets were stuck to a sample holder grid using Kapton tape and sealed within an aluminised Mylar bag under Ar atmosphere to prevent exposure of the samples to air during measurement. Ni K-Edge X-ray absorption near edge structure (XANES) spectra were collected. An X-ray beam was vertically collimated using a Pt-coated mirror before passing through the Si(111) double crystal monochromator. The X-ray beam was focused at the sample position using the Pt-coated groove of the monolithic double toroidal mirror. High-order harmonics in the incident beam were eliminated by using two dedicated Pt-coated mirrors operating at 7 mrad incidence angle. X-ray absorption spectra were measured in the vicinity of the Ni K-edge (∼8.3 keV). X-ray absorption was calculated using the Beer–Lambert law comparing the incident and transmitted X-ray intensities recorded by the first and second ionization chambers placed before and after the sample, respectively. Additional measurements across a nickel foil, using the second and third ionization chambers, provided reference data for energy calibration. Data processing was performed using the Demeter software package.^20^ For the normalization of the data, a pre-edge range between −150 and −30 eV from the edge center was fit with a straight line, and the post-edge region between 150 and 484 eV was fit with a quadratic polynomial. Data were transformed from *E*-space to *k*-space *via* the transform:

1 $$k=\sqrt{\frac{2m_{e}}{\hbar^{2}}\left(E-E_{0}\right)}$$

where *m*_e_ is the mass of the electron, *E*_0_ is the energy position of the absorption edge, and *E* is the energy of the measured photon.

Edge energy was estimated *via* fitting of a Gaussian peak to the first derivative of absorption with respect to measured energy (SI Section S-2, Figure S3), with the energy of the maximum taken to be the Ni K-edge energy. The stated error bars are then an indicative estimate only, representing half the distance between recorded energy points.

XAS was carried out on cast cathode films for this work. The X-ray absorption near edge structure data demonstrated shifting of the Ni K-edge as a function of desodiation (increasing oxidation state with sodium removal) are reported in SI Section S-2 and Figure S2.

### Synchrotron Powder X-ray Diffraction

SXRD experiments were carried out at beamline I11 at Diamond Light Source, U.K.^17^ Samples were measured in 0.5 mm diameter borosilicate glass capillaries, sealed with epoxy glue. The samples were measured using the Multianalyzer Crystal (MAC) detector, or Mythen II Position Sensitive Detector (PSD) as indicated in the text, at energies/wavelengths of 15 keV/λ ≈ 0.827 Å, refined against a Si standard. Measurements using the MAC detectors were collected at a step-size of 0.001°, and analyzed after rebinning at 0.010°. All reported measurements were collected at room temperature (approximately 25 °C).

*Operando* SXRD was collected using the Mythen II Position Sensitive Detector, at energies/wavelengths of 25 keV/λ = 0.494 Å as refined against a Si standard. Each data slice was combined from 4 × 30 s acquisition scans collected approximately every 12 min throughout electrochemical cycling.

Unfortunately, due to an issue with commissioning of the new position sensitive detector between different experimental sessions at the Diamond I11 beamline, there is significantly more noise in the more recently collected diffraction pattern for the *ex situ* O‴3-Na_1/3_NiO_2_, by comparison to the *ex situ* P″3-Na_1/2_NiO_2_ and O″3-Na_2/5_NiO_2_ and *operando* data sets.

### Neutron Powder Diffraction

Neutron powder diffraction measurements were obtained using the WISH diffractometer at the ISIS Neutron and Muon Source, U.K., which provides high signal-to-noise in the region where superstructure peaks are expected.^15^ Within a He-filled glovebox, samples were loaded into 5 mm internal diameter vanadium cans, and sealed with indium wire to prevent air/moisture exposure. The cans were loaded onto a sample changer, which was placed within the instrument sample tank under vacuum. Data were collected across all 5 pairs of instrumental banks (average 2*θ* of bank pairs 1_10 = 27.0°, 2_9 = 58.33°, 3_8 = 90.00°, 4_7 = 121.66°, 5_6 = 152.82°), on the samples containing ∼100 mg of Na_*x*_NiO_2_, and ∼43 mg of carbon (the cathode mixture, 70:30 active cathode:conductive carbon, see SI Section S-3 and Table S1 for more information), for approximately 4 h to ensure suitable signal-to-noise ratio for distinguishing the observed superstructure peaks. All reported measurements were collected at room temperature (approximately 25 °C).

### ISODISTORT + TOPAS Academic Superstructure Refinements

Rietveld and Pawley refinement of XRD/NPD data were carried out using TOPAS-Academic V7.^21–23^ Combined refinements of SXRD and NPD data (WISH TOF diffractometer banks 2_9, 3_8, 4_7, and 5_6) with data weighted such that the total contributions of the one XRD pattern and four NPD patterns were equal.^15^

XRD pattern backgrounds of P″3-Na_1/2_NiO_2_, O″3-Na_2/5_NiO_2_, and O‴3-Na_1/3_NiO_2_ were fit with Chebyshev polynomials with 53, 66, and 66 terms, respectively. Contributions to XRD peak shapes from the beam profile and instrument were from the beam were accounted for using a Thompson-Cox-Hastings pseudo-Voigt peak shape, the parameters of which were refined against a measured Si standard.^24^ Sample contributions to peak shapes were modeled using Lorentzian and Gaussian crystallite size parameters, and Stephens’-type monoclinic strain broadening.^25^

Instrumental contributions to peak shapes (DIFC, DIFA, ZERO, and tauf_1)^26^ in the NPD data were first fit to a measured NaCAlF standard. DIFC, initially 0.0, was later allowed to refine for all but the highest resolution bank, allowing for small differences in sample position within the instrument, the ZERO (which accounted for timing signal differences and finite response times in electrical components of the instrument) and tauf_1 (used in the moderator correction) were kept constant.^26^ These instrumental parameters were incorporated into TOF Lorentzian and Gaussian crystallite size and strain parameters, in addition to a TOF_2FP_Voigt peak shape. Further, Stephens monoclinic strain broadening was implemented using a custom TOPAS macro we developed for TOF NPD, as described in SI Section S-4. The complex nature of the NPD pattern backgrounds necessitated the use of a user-defined background using the bkg_file­() macro in TOPAS-Academic. A measured background scan of a vanadium can containing only the conductive carbon was input, with a scalar variable to increase or decrease its contribution appropriately for each sample data set (since this is only present as approximately 30% of the sample). A Chebyshev polynomial was included to improve the background fit.

Starting models for the Na_*x*_NiO_2_ supercells were generated either using structures reported from the literature or, when not available, using ISODISTORT.^27,28^ For O″3-Na_2/5_NiO_2_ and O‴3-Na_1/3_NiO_2_ it was possible to index the superstructure peaks observed using a *C*2*/m* space group, in agreement with the reported space group symmetries of related compounds with comparable Na concentrations (Na_2/5_CrO_2_,^29^ Na_1/3_CoO_2_).^14^ The reported Na_2/5_CrO_2_ structure did not contain an expanded unit cell with discrete vacancy ordering, thus the starting *b-*lattice parameter value of this cell was increased to reflect the expected 5-fold increase. The reported structure of Na_1/3_CoO_2_ was a supercell containing a 3 × *b* expansion (relative to the NaCoO_2_ parent unit cell), which was used as the starting point for the refinement of O‴3-Na_1/3_NiO_2_. For P″3-Na_1/2_NiO_2_ and O″3-Na_2/5_NiO_2_, ISODISTORT “method 2” was used (general method, search over specific *k* points), specifying the *k* point LD, k2 (0,*b*,0), with *b* = 1/2 and 2/5 respectively. As in the case of P′3-Na_2/3_NiO_2_ (using a prismatic proxy cell for the parent NaNiO_2_ structure),^12^ the irreducible representation LD2, k2t2, order parameter direction P1 and *k* point: (0, 1/2, 0) produced a prismatic cell with suitable expansion and space group symmetry (*P*2_1_ */m*) for the P″3-Na_1/2_NiO_2_ starting structure. Using the O′3-NaNiO_2_ parent structure, the irreducible representation LD1, k2t1, order parameter direction P1 and *k* point: (0, 2/5, 0) produced an octahedral cell with suitable expansion and space group symmetry (*C*2*/m*) for the O″3-Na_2/5_NiO_2_ starting structure. The three starting structures described were used as starting points for the Rietveld refinements, with lattice parameters substituted with those of the cells obtained *via* Pawley fitting.

The van Vleck mode analysis was carried out using the Python-based VanVleckCalculator software.^30,31^

## Results

### Electrochemical Synthesis of Desodiated Phases

The pristine active cathode material (O′3-NaNiO_2_) was synthesized as described in the Experimental Methods section, with identity and phase purity confirmed by Rietveld refinement of the previously reported *C*2*/m* structure against SXRD data.^12^ Prior to electrochemical preparation of the Na_*x*_NiO_2_ phases for *ex situ* experiments, complete charge–discharge cycles or a complete charge were measured using a large 1″ diameter Swagelok cell containing ∼100 mg NaNiO_2_ as the positive electrode. This showed the expected behavior and allowed the appropriate cutoff voltages for each phase to be selected (Figure 1). Samples of P″3-Na_1/2_NiO_2_, O″3-Na_2/5_NiO_2_, and O‴3-Na_1/3_NiO_2_ were isolated during the first charge in three separate experiments using fresh NaNiO_2_ electrodes charged to 3.25, 3.43, and 4.00 V vs Na, respectively, followed by voltage holds until the measured current was observed to drop to ∼0 mA (voltage held for 100 h). The exact final capacities at the end of the voltage holds were 116.4, 151.6, and 157.1 mAh.g^–1^. Assuming 100% Coulombic efficiency this is equivalent to Na^+^ contents (per formula unit) of 0.51, 0.36, and 0.33 for P″3-Na_1/2_NiO_2_, O″3-Na_2/5_NiO_2_, and O‴3-Na_1/3_NiO_2_, respectively. These Na contents are approximately commensurate with the electrochemistry shown in Figure 1, with slight differences from exact fractional values obtained in coin cell measurements being assigned to the inconsistent nature of the large Swagelok cells, errors associated with the weighing of powder mixtures vs loss of some powder in assembly of the cells and potentially parasitic side reactions. The fractional values given above are confirmed here *via* the structural refinements (see below).

**Figure 1:**
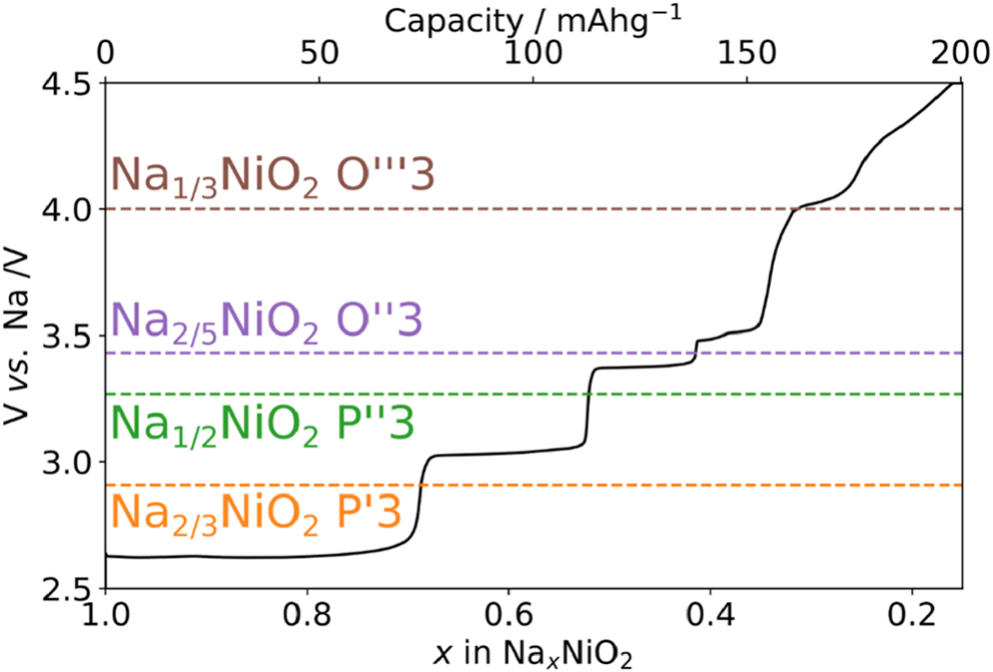
First electrochemical charge profile (C/100, cycled up to an upper cutoff voltage of 4.5 V) of NaNiO_2_ (black line), with voltage cutoff points used to prepare the *ex situ* samples overlaid (colored dashed lines). Note the P′3-Na_2/3_NiO_2_ phase was studied in previous work.^12^ Capacity is shown above and Na-content below (calculated assuming 100% Coulombic efficiency).

To ensure that we have captured all phases present in the electrochemistry, *operando* SXRD during electrochemical charging of a cell containing 24.4 mg active cathode material was performed as discussed further below. In addition to the expected stable phases from *ex situ* analysis, two metastable/transient phases (P‴3-Na_1/2<*x*<2/3_NiO_2_, and O″3δ-Na_1/3<*x*<2/5_NiO_2_) were additionally observed.

To confirm redox activity of Ni^3+^ → Ni^(3+δ)+^, X-ray absorption spectroscopy measurements were carried out on *ex situ* samples. X-ray absorption near edge spectroscopy (XANES) demonstrates a shifting of the Ni K-edge to higher energy across the series, confirming oxidation of Ni^3+^ as a function of desodiation (SI Section S-2, Figure S2).

### Combined Rietveld Refinement of Powder Diffraction Data

Since structural solutions are required before we can model the full *operando* data, we started with the analysis of *ex situ* XRD data. The SXRD patterns of the P″3-Na_1/2_NiO_2_, O″3-Na_2/5_NiO_2_, and O‴3-Na_1/3_NiO_2_ phases are in agreement with prior reports and indicate that phase pure samples with a single Na_*x*_NiO_2_ composition and no other impurity phases are present (see SI Section S-5 for more info).^13,14^ The layered (001) peak, corresponding to the interlayer spacing, was observed to shift to lower Q (higher *d*, increased interlayer spacing) with decreasing sodium concentration across the series (SI Section S-5 Figure S4) in both the *ex situ* SXRD and NPD. In all Na_*x*_NiO_2_ samples, superstructure peaks were observed in the same range as seen in prior XRD studies.^13,14^ As expected, the superstructure peaks in NPD have different intensities to the XRD patterns (Figure 2) due to the different scattering cross sections of the elements in the two experiments.

**Figure 2:**
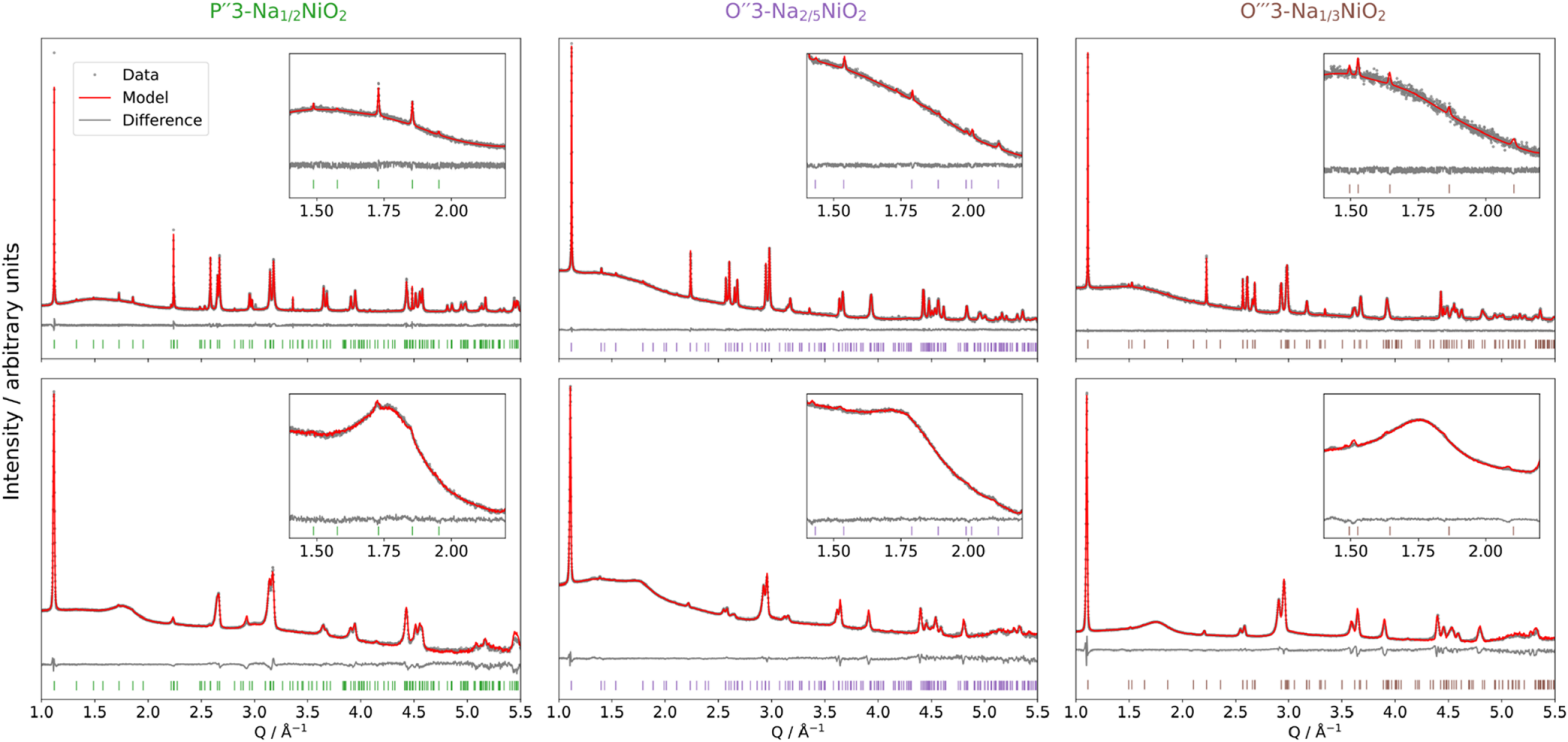
Combined Rietveld refinement against SXRD (top) and NPD (bottom) (average *2θ* of bank pairs 58.33°). Tick marks are displayed below for each phase: P″3-Na_1/2_NiO_2_ (green), O″3- Na_2/5_NiO_2_ (purple), O‴3-Na_1/3_NiO_2_ (brown). *R*_wp_/χ^2^ = 1.901%/1.111, 1.230%/2.829, 2.175%/5.652 respectively. Data collected at room temperature (approximately 25 °C). For SXRD, the square root of intensity is plotted on the *y*-axis for visual clarity. Insets show the superstructure peak region. Note increased noise in the SXRD pattern of O‴3-Na_1/3_NiO_2_ due to an update to the I11 PSD detector between dates of measurements. Additionally, the refinement showing good fit of the model O‴3-Na_1/3_NiO_2_ structure to the superstructural peaks in the *operando* data is given in SI Section S-9 Figure S8. This discrepancy is discussed further in the following text. Full structural details for each phase are given in SI Section S-6 Tables S2–S4.

We were able to index all peaks in the Na_*x*_NiO_2_ diffraction patterns, including the superstructure peaks, using supercells of the NaNiO_2_ parent structure, with commensurate expansions in the *b-*lattice direction. The observed expanded unit cells (sublattices) are, respectively: [1 × 2 × 1], [1 × 5 × 1], and [1 × 3 × 1] expansions for P″3-Na_1/2_NiO_2_,^13,14^ O″3-Na_2/5_NiO_2_ ^13^ and O‴3-Na_1/3_NiO_2_,^14^ consistent with prior work. Structural models were generated to use as initial models for quantitative structural analysis, as described in the Experimental Methods section.

In our initial analysis we refined Na site occupancies on all allowed Na positions within the cell (with unconstrained Na compositions). In all cases we identified full occupancy of a single Na site with other sites vacant, giving compositions of P″3-Na_1/2_NiO_2_, O″3-Na_2/5_NiO_2_, and O‴3-Na_1/3_NiO_2_. These values are consistent with the Na contents determined *via* the electrochemistry. In subsequent refinements, the occupancies of the singly occupied Na site in each phase were fixed to 1, and the remaining possible Na site occupancies were fixed to 0. From the starting structures, refinement of the allowed atomic coordinates for Ni and O and the occupied Na site in each structure facilitated good fits of the refined models to the data, with all peaks including the superstructure peaks in both the SXRD and neutron diffraction data well modeled (Figure 2). The structures obtained are discussed in detail below.

It is important to note that the structure used to model O‴3-Na_1/3_NiO_2_ appeared to add additional superstructure peak intensity, which was not present in the *ex situ* SXRD and NPD diffraction data, resulting in worse goodness of fit parameters (Figure 2, inset), but which was present in the *operando* SXRD data set (see below, and SI Section S-9, Figure S8 for more info). We also note that the *ex situ S*XRD data collected for this sample had higher noise than the P″3-Na_1/2_NiO_2_ and O″3-Na_2/5_NiO_2_ samples due to issues with the detector, further contributing to a higher *R*_wp_ than the refinements for the other two phases.

### Sequential Rietveld Refinement of *Operando* SXRD Data

Having determined the major phases in the Na_*x*_NiO_2_ phase diagram, we were then able to carry out quantitative analysis of the *operando* SXRD data using sequential Rietveld refinements. This analysis provided further insight into structural evolution during charge, including weight percentages of all phases present as a function of state of charge, functioning effectively as an electrochemical reaction coordinate.

The electrochemistry proceeds as expected. From OCV (∼2.21 V), a sharp rise in voltage occurs (from OCV to ∼2.7 V, Figure 3a) corresponding to the first biphasic reaction (O′3-Na_1_NiO_2_ → P′3-Na_2/3_NiO_2_). Following this, there are further voltage plateaus (∼3.0, ∼3.4 V), corresponding to the P′3-Na_2/3_NiO_2_ → P″3-Na_1/2_NiO_2_, P″3-Na_1/2_NiO_2_ → O″3-Na_2/5_NiO_2_ reactions, respectively. There is then a slightly sloping region 3.45 – 3.50 V, where a new phase is identified, which we label O″3δ-Na_1/3<*x*<2/5_NiO_2_. The voltage then finally rises sharply toward 4.5 V as the final O‴3-Na_1/3_NiO_2_ phase is produced. Beyond this, electrolyte degradation and/or the O1–Na_0<*x*<1/3_NiO_2_ phase may in principle form,^32^ but the O1–Na_0<*x*<1/3_NiO_2_ phase is not seen in the cycling range used here. As per prior work, a small amount of a phase seen previously at the end of discharge (and labeled O⁗3-Na_2/3<*x*<1_NiO_2_)^16^ is seen in the as-cast electrode. This is likely due to partial desodiation from reaction of sodium with either trace moisture in the NMP solvent used in casting or in the propylene carbonate electrolyte.

**Figure 3:**
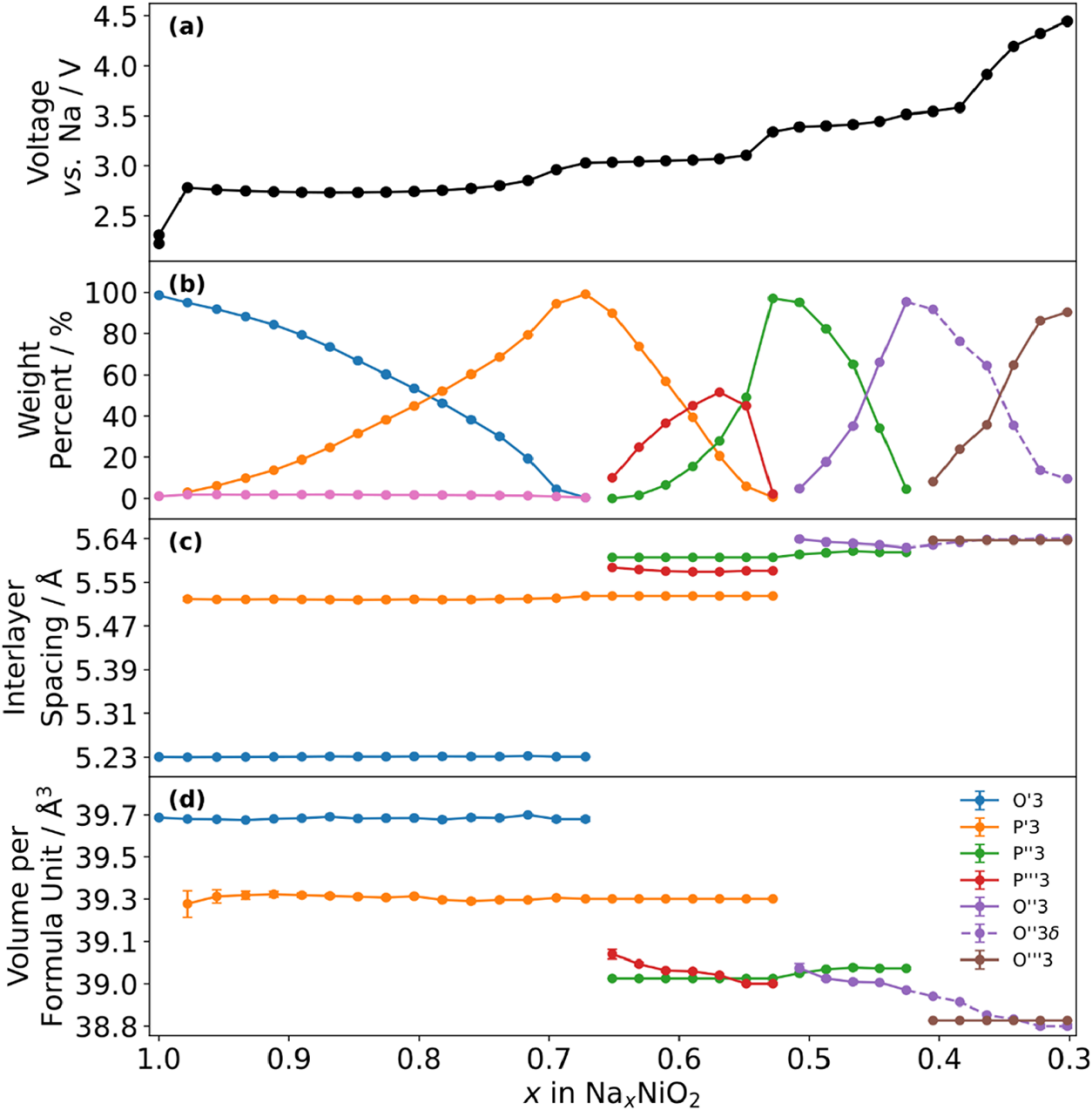
(a–d): (a) Electrochemical voltage profile of the *operando* cell, (b) weight percent, (c) interlayer spacing, and (d) volume per formula unit, as a function of sodium content during electrochemical charge, calculated *via* sequential Rietveld refinement of *operando* SXRD. O⁗3-Na_2/3<*x*<1_NiO_2_ (pink) is not included outside of weight percentage as a structural solution for this phase is not reported in this work, and a proxy cell based on O′3 is used to fit the single peak present in the data.

In addition to the structures identified from our *ex situ* experiments, two extra transient phases that could not be isolated *ex situ* are also observed. The first is another prismatic phase (P‴3-Na_1/2<*x*<2/3_NiO_2_) observed at the onset of the P′3-Na_2/3_NiO_2_ → P″3-Na_1/2_NiO_2_ transition (∼3.0 V) (see SI Section S-7 for more info). This P‴3-Na_1/2<*x*<2/3_NiO_2_ phase is only present when both the other phases are also present. It has intermediate interlayer spacing, and a volume closer to P″3-Na_1/2_NiO_2_, which contracts further (in the *ab-*plane) as a function of Na content (Figure 3c,d). We note that the superstructure peaks of both the P′3-Na_2/3_NiO_2_ and P″3-Na_1/2_NiO_2_ structures disappear while this phase is present and are replaced by a large broad feature (most prominent in the slice at ∼3.05 V, Figure S5). Since the rest of the observed pattern remains sharp, the emergence of a broad superstructure reflection (FWHM = 0.39[5]° vs ∼0.0408[3]° for P′3-Na_2/3_NiO_2_ superstructure peaks) suggests formation of a distinct, discrete Na^+^/vacancy ordering with shorter coherence length (Scherrer crystallite size ∼coherence length = 65[8] Å vs ∼490 Å for P′3-Na_2/3_NiO_2_ superstructure peaks, Figure S6). Attempts to isolate this phase *ex situ* to obtain a full structure solution resulted in samples that disproportionated into P′3 and P″3, suggesting that this is a metastable phase. We hypothesize that this transient phase buffers the transition from one phase to another, since it is associated with a volume that is intermediate between the two stable prismatic phases, which have large differences in their interlayer spacings and cell volumes. A full structural solution is beyond the scope of the current work and is presently being investigated.

The second new phase is observed in the *operando* SXRD data for 1/3 < *x* < 2/5. A “continuous shifting” of the layered (001) peak was observed at similar contents in prior *in situ* XRD studies of electrochemical cycling in Na_*x*_NiO_2_, which was described in terms of a solid-solution type phase.^6,33^ In this composition range, the data can be well modeled by assuming a solid solution-type reaction and allowing the lattice parameters of an O″3-Na_1/3_NiO_2_ based proxy phase to vary in the range between the limits of the O″3-Na_2/5_NiO_2_ and O‴3-Na_1/3_NiO_2_ end phases, with Na being lost continuously. No new superstructure peaks were observed to form in this region, rather those of O″3-Na_2/5_NiO_2_ disappear, before those of O‴3-Na_1/3_NiO_2_ emerge (Figure S7b); this suggests, however, that this is not a simple solid solution and that a degree of cation ordering persists. However, the data quality was not sufficient to allow further analysis. The lack of new unique superstructure peaks is in contrast to the broad, additional superstructure peaks demonstrated by the P‴3-Na_1/2<*x*<2/3_NiO_2_ phase (see SI Section S-8 for more information).

At the end of charge and for the O‴3-Na_1/3_NiO_2_ phase, we note that the allowed reflections previously absent from the *ex situ* data (Figure 2) are clearly visible in the *operando* SXRD data, and are well-fit by the model (SI Section S-9, Figure S8). However, the reported *R*_wp_ for the *operando* SXRD fit at this composition is notably higher (*R*_wp_ = 8.232% vs 2.175%) despite the better visual fit to the superstructure peak region. This can be attributed to a number of factors. First, the more complex background characteristic of *operando* data (containing conductive carbon, PVDF binder, and glass fiber separator), and the necessity to exclude regions of the data where steel, aluminum, and sodium peaks are present from the *operando* cell components. Second, only a single SXRD data set is fit, as opposed to 5 data sets (1 SXRD, 4 NPD) in the combined refinement. As the SXRD measurements are significantly less sensitive to the O positions, and we are able to achieve good fits (accounting for all observed reflections) using the combined refinement derived structure, we elect not to refine the O, Na, and Ni atomic positions, so as to not introduce additional unnecessary degrees of freedom to the refinement. This marginally reduces the quality of the fits (by comparison to the combined refinements), but does not introduce additional unwarranted structural uncertainty to the refinements. Despite these limitations, the structure determined for O‴3-Na_1/3_NiO_2_ fits the *operando* pattern, including the superstructure peaks, confirming the presence of the 3 × *b*_O′3_ unit cell expansion required to accommodate the Na^+^/vacancy ordering. We hypothesize that the discrepancy between the *ex situ* and *operando* SXRD data may be due to relaxation of the sample, or reaction during handling. Given the highly hygroscopic nature of the desodiated layered phases, it is not unreasonable that the most desodiated sample (having the largest interlayer spacing), would be the most facile toward reaction/degradation.

### Structures of Electrochemically Desodiated Na_*x*_NiO_2_

The room-temperature structures determined for the Na_*x*_NiO_2_ phases are shown in Figure 4a–e,i–iii and the details discussed below. For completeness an overview of the previously reported O′3-NaNiO_2_ and P′3-Na_2/3_NiO_2_ is also included. Cell parameters for each phase are provided in Table 1, a summary of the general features in Table 2, and specific structural details in Table 3. All the following charges for Ni^x+^ sites were derived from BVS calculations.

**Table 1:** Sodium Content, Cell Parameters, and Voltage Cutoffs for the Desodiated Phases Reported in this Study^a^

	Na content			Cell Parameters								
Phase	Han *et*. *al*.^6^	Current Study	Space Group	*a* (Å)	*b*_super_ (Å)	*b*_sub_ (Å)	*c* (Å)	β (°)	*a*/*b* Ratio	Interlayer Distance/Å	Vol. per F.U./Å^3^	Voltage/V
O′3- NaNiO_2_ ^9^	Na_1.00_NiO_2_	Na_1.00_NiO_2_	*C*2*/m*	5.3192(2)	NA	2.8451(1)	5.5826(4)	110.449(2)	1.8701(7)	5.2308(4)	39.58(4)	NA
P′3- Na_2/3_NiO_2_ ^12^	Na_0.70_NiO_2_	Na_2/3_NiO_2_	*P*2_1_ */c*	4.97153(7)	8.58914(11)	2.86305(4)	5.74466(3)	105.9132(10)	1.73637(3)	5.5175(4)	39.235(8)	2.90
P″3- Na_1/2_NiO_2_	Na_0.55_NiO_2_	Na_1/2_NiO_2_	*P*2_1_ */m*	4.91947(9)	5.66324(11)	2.83162(11)	5.82204(15)	105.63782(4)	1.73640(9)	5.6055(18)	39.050(2)	3.25
O″3- Na_2/5_NiO_2_	Na_0.45_NiO_2_	Na_2/5_NiO_2_	*C*2*/m*	4.94085(4)	14.03925(13)	2.80785(3)	5.86783(4)	106.51831(10)	1.75930(2)	5.6352(4)	39.023(4)	3.43
O‴3- Na_1/3_NiO_2_	Na_0.37_NiO_2_	Na_1/3_NiO_2_	*C*2*/m*	4.93408(11)	8.38899(18)	2.79633(6)	5.90396(12)	107.142(3)	1.7653(6)	5.6362(15)	38.920(2)	4.00

a Sodium contents reported previously, and known cell parameters for the O′3 phase are included for comparison. Note that *a* and *c* have been swapped by comparison to our prior report of the P′3 structure for convenience of comparison with the new structures produced in the non-conventional monoclinic setting. In this case, all interlayer distances are calculated as c sin­(β). Ratio of *a*/*b* given as a metric of in-plane monoclinic distortion (see SI Section S-14 for more information). *b*_super_ is the *b*-lattice parameter of the supercell expansion, whilst *b*_sub_ is the value for an equivalent, unexpanded cell given for ease of comparison between each structure. Where not applicable (NA) is given, this is due to there being no supercell for this structure.

**Table 2:** Summary of Structural Properties of the Reported Phases

	O′3-NaNiO_2_	P′3-Na_2/3_NiO_2_	P″3-Na_1/2_NiO_2_	O″3-Na_2/5_NiO_2_	O‴3-Na_1/3_NiO_2_
Space Group	*C*2*/m*	*P*2_1_/*c*	*P*2_1_/*m*	*C*2*/m*	*C*2*/m*
Supercell Expansion with Respect to NaNiO_2_	(1 × 1 × 1)	(1 × 3 × 1)	(1 × 2 × 1)	(1 × 5 × 1)	(1 × 3 × 1)
Na^+^/Vacancy Ordering	No Vacancies	Zigzag	Chains	Pairs	Isolated
TM Site Ordering	NA	Honeycomb	Stripe	Pseudo-Honeycomb	Honeycomb
TM Charge Ordering	NA	Honeycomb	None	Mirrored Zigzag	Honeycomb
Alkali Metal Site Connectivity	6 Edge-Sharing	3 Edge-Sharing	2 Edge-Sharing	1 Edge-Sharing	No Na–Na Interactions
Similar Phase in Literature	NA	Na_2/3_Cu_1/3_Mn_2/3_O_2_ ^44^	Na_1/2_CrO_2_ ^36^	Na_2/5_CrO_2_ ^29^	Na_1/3_CoO_2_ ^14^

**Table 3:** Structural Data of the Reported Phases and Benchmark Structures from Literature^a^

	O′3 (Na_1_)^9^	P′3 (Na_2/3_)^12^		P″3 (Na_1/2_)		O″3 (Na_2/5_)			O‴3 (Na_1/3_)		α-NaFeO_2_ ^45^	LiCoO_2_ ^46^	Na_0.677_CoO_2_ ^47^	IE-LiNiO_2_ ^48^
	Ni_(1)_ *2a*	Ni_(1)_ 2*a*	Ni_(2)_ 4*e*	Ni_(1)_ 2*a*	Ni_(2)_ 2*e*	Ni_(1)_ 2*a*	Ni_(2)_ 4*g*	Ni_(3)_ 4*g*	Ni_(1)_ 2*a*	Ni_(2)_ 4*g*	Fe_(1)_ 3*b*	Co_(1)_ 3*b*	Co_(1)_ 2*a*	Ni_(1)_ 2*d*
Average *TM*-O Bond Length /Å	1.988	1.985(11)	1.928(6)	1.922(10)	1.920(10)	1.920(6)	1.879(4)	1.919(4)	1.928(6)	1.888(4)	2.029	1.921	1.909	1.9706
*TM*O_6_ Octahedral Volume /Å^3^	10.24	10.16	9.36	9.29	9.33	9.38	8.57	9.25	9.29	8.81	10.91	9.37	9.12	10.13
BLDI /a.u.	0.052	0.051(5)	0.031(4)	0.019(5)	0.029(8)	0.017(3)	0.015(3)	0.029(1)	0.018(3)	0.034(3)	0.000	0.000	0.000	0.005
Quadratic Elongation /a.u.	1.018	1.020(11)	1.015(6)	1.014(11)	1.009(10)	1.005(6)	1.022(4)	1.014(4)	1.019(7)	1.014(4)	1.013	1.006	1.011	1.005
BAV /^o2^	40.128	46.7(49)	46.6(24)	41.4(58)	26.5(41)	13.9(16)	69.8(23)	41.3(20)	61.1(32)	40.6(22)	43.352	19.731	36.924	15.974
Site BVS	2.95	3.00(1)	3.43(6)	3.45(11)	3.51(10)	3.46(6)	3.90(4)	3.51(4)	3.38(7)	3.87(4)	2.84	2.78	2.88	2.96
Average BVS	2.95	3.29(3)		3.48(6)		3.66(2)			3.71(2)		2.84	2.78	2.88	2.96
Nominal TM Charge	3.00	3.33		3.50		3.60			3.67		3.00	3.00	3.33	3.00

a Included as points of comparison for layered sodium *TM*Os with no cooperative JT ground state (α-NaFeO_2_), desodiated analogues of these (Na_0.677_CoO_2_), the lithium-containing analogue with related bulk cooperative JT distortion (IE-LiNiO_2_), and finally LiCoO_2_ to contrast as an Li-containing analogue with no ground state JT distortion.^45–47^

**Figure 4:**
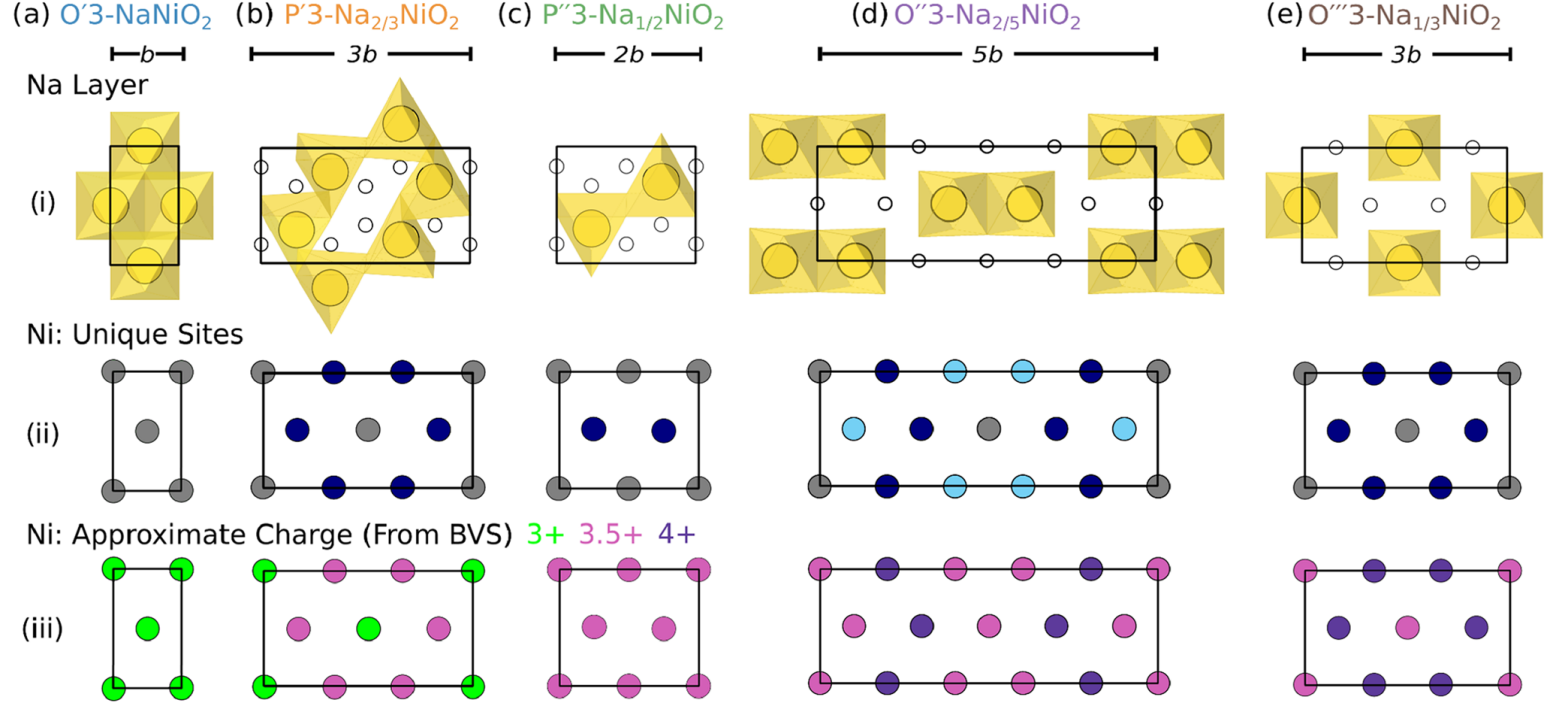
(a–e),(i–iii): Room temperature structures of the different Na_*x*_NiO_2_ phases (left to right *x* = 1, 2/3, 1/2, 2/5, 1/3). Top: Na interlayer vacancy ordering (Na represented by yellow circles, vacant octahedral/prismatic sites in Na layer represented by white circles). Middle: Ni crystallographic sites (Ni_(1)_ gray, Ni_(2A)_ dark blue, Ni_(2B)_ light blue). Bottom: Ni charge ordering (oxidation states close to 3+ in green, 3.5+ in pink, 4+ in purple). 3*a* × 3*b* × 1*c* expansions of the Na layer are given in SI Section S-10 Figure S9 to illustrate the vacancy ordering nomenclature. Note that in (i) Na layer, only vacant octahedral/prismatic sites within the triangular/honeycomb Na lattices respectively are illustrated.^43^ Additional crystallographically-allowed Na sites (generated with respect to the parent structures) are omitted to clarify the Na lattices in O3/P3 layered structures.

#### O′3-NaNiO_2_

O′3-NaNiO_2_ has *C*2*/m* space group symmetry, containing a single crystallographic site for Na (2*a*), Ni (2*a*), and O (4*i*) (Figure 4ai/ii). By contrast to the archetypal O3-type Ni-rich Li cathode material (LiNiO_2_),^34^ NaNiO_2_ exhibits a cooperative JT distortion in its ground state. This results in symmetry lowering from the ideal *R*3̅*m* rhombohedral symmetry, to *C*2*/m* monoclinic symmetry due to the elongation in one of three octahedral axes, and contraction of the remaining two axes, of the NiO_6_ octahedra. The Ni layer formally contains only d^7^ (t_2g_^6^, e_g_^1^) Ni^3+^ ions (Figure 4aiii), each in a tetragonally distorted NiO_6_ octahedron, with their elongated axis pointing in the same direction to form a collinear JT ordering arrangement.^9,35^ There is no Na^+^/vacancy ordering, as the Na interlayer is fully occupied (Figure 4ai). Each NaO_6_ octahedron has 6 edge sharing interactions with other octahedral Na sites.

#### P′3-Na_2/3_NiO_2_

The P′3-Na_2/3_NiO_2_ structure has *P*2_1_ */c* space group, and forms *via* a layer-glide transition (perpendicular to the *c*-axis), and the NaO_6_ units are now prismatically coordinated. Its unit cell features a 3-fold expansion in the *b-*lattice direction by comparison to the parent NaNiO_2_ structure. The expanded unit cell results in two distinct Ni sites present in a 1:2 ratio (2*a*, 4*e)*, three O sites (all 4*e*), and 3 crystallographically-allowed Na sites (all 4*e*). A single Na site (4*e*) is fully occupied with the others vacant (Figure 4bi/ii), the resulting Na^+^/vacancy ordering forming a “zigzag” pattern in the Na interlayer (Figure 4bii). This arrangement minimizes Na^+^-Na^+^ repulsive electrostatic interactions, as there are no face-sharing polyhedra, with 3 edge-sharing interactions instead. The two Ni sites correspond to one which is more reduced (Ni_(1)_, formally Ni^3+^), and one which is more oxidized (Ni_(2)_, approximately Ni^3.5+^). (Figure 4biii). The average Ni oxidation state is effectively Ni^3.33+^, as expected for *x* = 2/3. The less oxidized (higher volume) Ni_(1)_^3+^ sites are surrounded by more oxidized (lower volume) Ni_(2)_ sites in a honeycomb charge-ordering and the larger Ni_(1)_O_6_ octahedra are arranged such that the longer Ni–O bonds point toward vacancies in the Na layer, minimizing interlayer repulsive electrostatic interactions.

#### P″3-Na_1/2_NiO_2_

The Na_1/2_NiO_2_ phase has *P*2_1_ */m* space group symmetry, with a 2-fold expansion in the *b-*lattice direction in comparison to the parent NaNiO_2_ structure. The structure again possesses three crystallographically-allowed Na sites (2*e*, 4*f*, 4*f*), with a single preferentially occupied Na site (2*e*), and two vacant Na sites (both 4*f)*, in which Na^+^ ions in NaO_6_ units are prismatically coordinated by O^2–^ ions. There are two crystallographic Ni sites (2*a*, 2*e*), and three crystallographic O sites (2*e*, 2*e*, 4*f)* (Figure 4ci/ii). With respect to the Na^+^/vacancy ordering, this structure resembles the Na_1/2_CrO_2_ structure, with Na^+^/vacancies arranged in a so-called “edge-face” arrangement, in reference to the prismatic NaO_6_ units alternating between edge-sharing and face-sharing with the transition metal octahedra.^36^ The Na^+^ ions form “chains” sharing 2-edges with adjacent Na prisms (Figure 4ci, SI Section S-10 Figure S9). By comparison to P′3, this arrangement further minimizes intralayer Na^+^-Na^+^ electrostatic repulsions, by decreasing the number of prismatic edge-sharing interactions from 3 to 2. The two crystallographically-distinct Ni sites are arranged in alternating stripes of Ni_(1)_ (2*a*) and Ni_(2)_ (2*e*) sites (Figure 4cii), exhibiting oxidation states of Ni^3.45+^ and Ni^3.51+^, respectively, demonstrating a lack of physically meaningful charge separation across the two sites (Figure 4ciii).

#### P‴3-Na_1/2<*x*<2/3_NiO_2_

While it is not possible to isolate this metastable phase as a pure sample to facilitate a unique determination of its structure, the presence of a single unique broad superstructure feature suggests that there is a phase with a composition between that of P′3-Na_2/3_NiO_2_ and P″3-Na_1/2_NiO_2_, possibly *x* = 4/7, as this is an observed ordering in other layered Na cathodes.^37^ The broadness of the feature, when compared to other reflections, suggests Na^+^/vacancy ordering with short coherence length as observed in some rock salt cathode systems.^38^ Solving of the structure likely requires combined refinement of SXRD and NPD data, precluding a structure solution in this work. This phase can accommodate some Na nonstoichiometry as it has a volume that while initially close to P″3-Na_1/2_NiO_2_, contracts further (in the *ab-*plane) on charging eventually reaching that of P″3-Na_1/2_NiO_2_ (Figure 3c,d). No clear signature of an intermediate phase is seen electrochemically (Figures 1 and 3a).

#### O″3-Na_2/5_NiO_2_

Upon further desodiation to *x* = 2/5 the layer glide is reversed, and an O″3-Na_2/5_NiO_2_ phase with *C*2*/m* space group symmetry is now formed. This cell has a 5-fold expansion in the b-lattice with respect to the parent O′3-NaNiO_2_ structure. There are 3 crystallographically-allowed Na sites (2*d*, 4*h*, 4*h*), along with three crystallographic O sites (4*i*, 8*j*, 8*j)* and 3 Ni sites (2*a*, 4*g*, 4*g*) present in a 1:2:2 ratio (Figure 4dii). Again only a single Na site (4*h*) is occupied, the occupied Na sites forming “pairs”, with each NaO_6_ octahedra sharing a single edge with a second site, decreasing the number of edge-sharing interactions to one (Figure 4di). The Na layer bears similarities to the Na layer in the Na_2/5_CrO_2_ structure.^29^ However, in Na_2/5_CrO_2_, there is no expansion in *b* and all allowed Na sites are partially occupied with occupancies of 2/5 (i.e., there is site disorder, with overall Na concentration *x* = 2/5). The Ni sites in O″3-Na_2/5_NiO_2_ are arranged such that the Ni_(1)_ (2*a)* is surrounded by Ni_(2)_ and Ni_(3)_ sites (both 4*g*), forming a pseudohoneycomb arrangement (Figure 4dii). The Ni_(1)_ (2*a*) and Ni_(3)_ (4*g*) sites have similar charges (Ni^+3.46^ and Ni^+3.51^ respectively), while the charge on the Ni_(2)_ (4*g*) sites is much higher (Ni^+3.90^), resulting in an overall average Ni oxidation state of Ni^3.66+^ ≈ Ni^3.60+^ (consistent with electrochemically removing 3/5 Na^+^). This arrangement of Ni charges has been described as a “mirrored-zigzag”, with “zigzags” of highly oxidized Ni_(2)_ sites flanked on either side by lower oxidation state Ni_(1)_ and Ni_(2)_ sites (Figure 4diii). The Ni_(2)_ site has the shortest bond lengths and most contracted NiO_6_ octahedra present across all the desodiated phase structures reported here (Table 2, Figures 5, SI Section S-11 Figure S10). We note that Mock *et al*. reported a similar ordering for the lithium analogue (with slight off-site stoichiometry) O3-type phase Li_2/5_Ni_1.02_O_2_, corresponding to the “M” (monoclinic) phase in the electrochemical cycling of Li_*x*_NiO_2_, in their work. This phase shows the same patterning of “pairs” of occupied NaO_6_ octahedra, with single edge-sharing interactions.^39^

**Figure 5:**
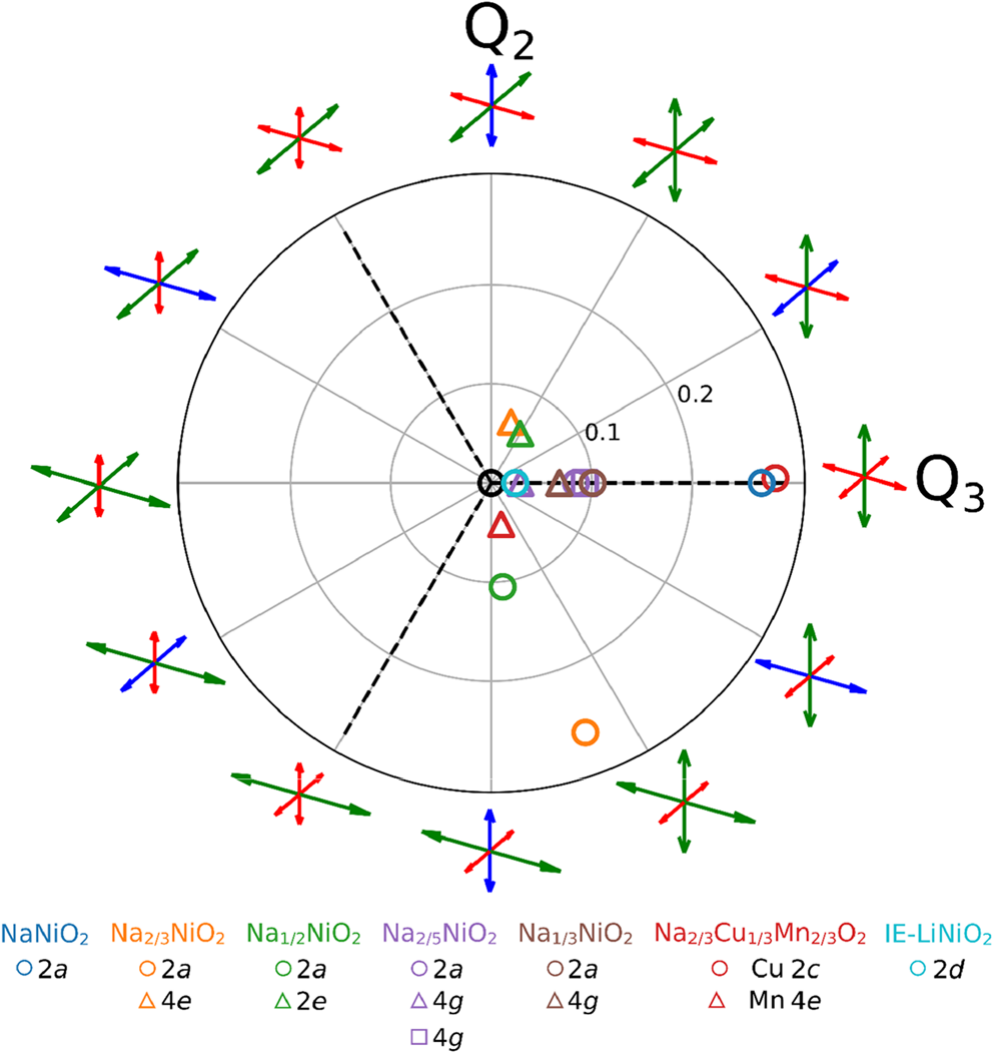
Polar plot of Q_2_/Q_3_ Van-Vleck octahedral distortion modes, illustrated by surrounding octahedral axes showing compressed (red), undistorted (blue), and elongated (green) axes. Marker colors denote desodiated phases O′3-NaNiO_2_ (light blue), P′3-Na_2/3_NiO_2_ (orange), P″3-Na_1/2_NiO_2_ (green), O″3-Na_2/5_NiO_2_ (purple), O‴3-Na_1/3_NiO_2_ (brown), and selected benchmark structures Na_2/3_Cu_1/3_Mn_2/3_NiO_2_ (dark red)­Ion-exchange synthesized LiNiO_2_,^48^ α-NaFeO_2_,^45^ LiCoO_2_,^46^ and Na_0.677_CoO_2_ ^47^ (all black circles) are zero, hence overlap at the origin in this plot. Shapes denote crystallographic sites Ni_(1)_ (circles), Ni_(2A)_ (triangles), and Ni_(2B)_ (square, note there is only one such site and it overlaps with the Ni_(1)_ site of the same phase).

#### O‴3-Na_1/3_NiO_2_

The Na_1/3_NiO_2_ phase, labeled O‴3, has *C*2*/m* space group symmetry and a 3-fold expansion in the *b*-lattice direction by comparison to the parent O′3-NaNiO_2_ structure. There are 3 crystallographically-allowed Na sites (2*c*, 2*d*, 4*h*), with a single fully occupied site (2*d*), two O sites (4*i*, 8*j)*, and 2 Ni sites (2*a*, 4*g)*, generating a honeycomb arrangement in the Ni layer (Figure 4eii). The 4i site is noticeably more reduced (Ni^3.38+^) compared to the 4g site (Ni^3.87+^), resulting in average Ni charge Ni^3.71+^∼Ni^3.67+^, commensurate with removal of 2/3 of the Na^+^ (Figure 4eiii). The Na octahedra are now isolated, with no edge- or face-sharing interactions with other occupied Na sites, minimizing Na^+^-Na^+^ electrostatic repulsions (Figure 4ei). With respect to the Na layer, O‴3 bears similarities to the Na_1/3_CoO_2_ structure.^14^

#### O″3δ-Na_1/3<*x*<2/5_NiO_2_

As discussed above, we observe a phase transition from an ordered O″3-Na_2/5_NiO_2_ phase to a solid solution O″3δ-Na_1/3<*x*<2/5_NiO_2_ phase (i.e., a phase that accommodates a degree of Na contents/does not have a fixed volume), before the formation of the ordered O‴3-Na_1/3_NiO_2_ phase. The voltage profile in this Na composition range is not flat and is consistent with one or more phases. Since the O″3δ-Na_1/3<*x*<2/5_NiO_2_ phase is not isolable *ex situ*, the high SNR SXRD/NPD measurements required to solve this phase are not possible at present. No discrete ordering observed in the present data (see Figure S7 for more information), suggesting that this solid-solution phase (having no well-ordered Na^+^/vacancy patterning) forms as a buffer between the two stable Na^+^ concentrations on either side, which have large differences in their volumes/interlayer distances. As with the P‴3-Na_1/2<*x*<2/3_NiO_2_ phase, the intermediate phases with no or partial ordering, are found between phases with the same layer stacking (P and O), so that the buffering only involves cation arrangements (and Ni oxidation state).

#### O1–Na_0<*x*<1/3_NiO_2_

In attempts to prepare samples of the O‴3-Na_1/3_NiO_2_, some samples were charged to 4.5 V (see SI Section S-12, Figure S7). However, on electrochemical cycling past 4.0 V, we begin to observe electrolyte degradation in the voltage curve, as well as the formation of an O1–Na_0<*x*<1/3_NiO_2_ phase (∼17 wt %), with a corresponding (001) peak with *d*-spacing ∼4.35 Å, suggestive of an irreversible layer shearing and collapse (SI Section S-12 Figure S11), akin to the O1–Na_0<*x*<1/3_NiO_2_ “NiO_2_”^40^ phase that forms at very high voltages in delithiated Li_x_NiO_2_.^41^ An O3- → O1-type transition has been reported at ∼4.0 V in 10% Ti doped NaNiO_2_, resulting in approximately 30% contraction of the interlayer spacing and associated particle cracking.^32^ We observe a similar magnitude contraction in the O1–Na_0<*x*<1/3_NiO_2_ phase (interlayer spacing [*c* sin­(β)] = 3.66(50) Å vs 5.23 Å in O′3-NaNiO_2_). However, since this phase forms *via* a process which occurs outside of the stable electrolyte window, it is not possible to reliably produce this phase electrochemically. Therefore, a rigorous structural solution, beyond the above approximation presented based on the atomic structure of the O1 phase in LiCoO_2_ (reported by Tarascon et al.)^42^ is beyond the scope of the present work.

## Discussion

By using a combined analysis of SXRD and NPD data we have determined the structures of each of the stable Na^+^/vacancy ordered phases that form during charge of O′3-NaNiO_2_ → P′3-Na_2/3_NiO_2_ → P″3-Na_1/2_NiO_2_ → O″3-Na_2/5_NiO_2_ → O‴3-Na_1/3_NiO_2_. The Na concentrations of these phases are confirmed both from electrochemistry (assuming 100% Coulombic efficiency), and crystallographically from the ratios of occupied sites in the Rietveld refined structures. Through our work we resolve some of the conflicting reports with respect to the sodium content of these phases.^13,14^

Our work demonstrates the need for both *ex situ* and *operando* studies of electrochemical systems. It is only through the *ex situ* studies combining NPD and SXRD that we are able to fully determine the structures adopted on cycling NaNiO_2_. The biphasic nature of most of the electrochemical phase transitions was confirmed through sequential Rietveld refinement analysis of the *operando* data. *Operando* experiments also allow us to identify transient phases that are not isolable *via ex situ* studies.

The two transient phases identified in our *operando* experiments have different characteristics. The more sodium-rich P‴3-Na_1/2<*x*<2/3_NiO_2_ phase has a broad, low intensity feature in the superstructure peak region, possibly reflective of a discrete ordering (*i*.*e*. 4/7) with low coherence length (see SI Section S-7 for more information). The sodium-poor metastable phase, O″3δ-Na_1/3<*x*<2/5_NiO_2_, is not observed to exhibit unique superstructural ordering, commensurate with prior hypothesis of a transition to a solid-solution in this region (see SI Section S-8 for more information).^6^ We note that with the time resolution limit of our current *operando* SXRD (∼12 min/∼0.02 *x* in Na_1–*x*_NiO_2_ between scans), we may not be sensitive to a case in which there are stable orderings at closely spaced (*x* < 0.02) concentrations, such as the notorious “devil′s staircase” of orderings seen in Na_*x*_MoO_2_ for 0.5 < *x* < 0.75 where 8 distinct superstructures were identified by *operando* SXRD in this compositional range.^49^ However, the signatures of these different phases were also clearly visible in the electrochemical voltage profiles, which is not the case for Na_*x*_NiO_2_, at least in the composition range Na_1/2<*x*<2/3_NiO_2_. Additionally, the observed broad, low-intensity feature is not reflective of a series of discrete orderings, which would result in unique superstructure peaks from each unique k-vectors for a series of ordered phases. We note that Mo–Mo bonds are formed within the Na_*x*_MoO_2_ phase space,^50^ providing another strong driving force for superstructure formation, beyond those relevant to the current Na_*x*_NiO_2_ system. Slower charge rates (such as C/20 or slower), and higher time-resolution measurements, would be required to investigate this thoroughly.

Having determined the atomic structures of the Na_*x*_NiO_2_ phases generated on electrochemical cycling, we now consider trends in behavior across the series. All the phases identified retain a layered structure with edge-sharing NiO_6_ octahedra. An increase in the interlayer spacing on removal of Na is observed (Figure 3c) as previously reported, and this is accompanied by a decrease in the volume per formula unit. We find that both average Ni–O bond length and average NiO_6_ octahedral volume decrease on desodiation,^6^ consistent with Ni oxidation (Figures 3d and 6) as observed by XANES (SI Section S-2 Figure S2). In contrast, the NaO_6_ polyhedra (octahedral or prismatic) increase in bond length as Na content is reduced. We ascribe this to decreased electrostatic repulsions in the Na layer as energy-raising edge-sharing interactions are minimized (see SI Section S-13 Figure S12 for more information on the structural evolution of Na interactions).

**Figure 6:**
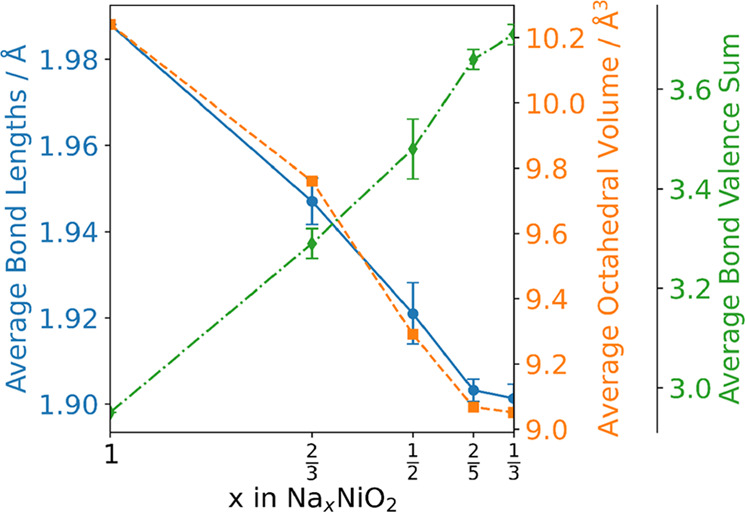
Average bond lengths (blue circles), weighted average octahedral volumes (orange squares), and average bond valence sum (green diamonds) for the NiO_6_ octahedra present in the Na_*x*_NiO_2_ phases.

With the exceptions of O′3-NaNiO_2_, where all Na sites are fully occupied and there is a single Ni^3+^ oxidation state, and the transient phases (P‴3-Na_1/2<*x*<2/3_NiO_2_, O″3δ-Na_1/3<*x*<2/5_NiO_2_), all structures display Na^+^/vacancy ordering. As sodium content decreases, the relative connectivity of the Na sites (octahedra or prisms) also decreases. The number of energy-raising edge-sharing interactions present is lowered from 6 (all edge-sharing) →3 → 2 → 1 → 0 across the series from O′3-NaNiO_2_ → P′3-Na_2/3_NiO_2_ → P″3-Na_1/2_NiO_2_ → O″3-Na_2/5_NiO_2_ → O‴3-Na_1/3_NiO_2_. The features of each desodiated structure (Figure 4 and Table 2) suggest that minimizing repulsive electrostatic Na^+^-Na^+^ interactions is a significant factor in stabilizing the vacancy-ordered phases, as has long been suggested in the related systems including Na_*x*_CoO_2_,^51–53^ and Na_*x*_VO_2_,^54^ with theory supported by DFT calculations suggesting that this stabilization is applicable across O1/O3/P3 layered cathode structures.^43^

We use BVS analysis to evaluate the changes in the oxidation state of Ni on desodiation. At higher Ni oxidation state, the bonding is likely to become more covalent and so this approach, which assumes purely ionic interactions, may not fully capture the changes. Nonetheless, it does enable a nominal oxidation for each Ni site to be obtained. Our analysis indicates that in the P′3-Na_2/3_NiO_2_, O″3-Na_2/5_NiO_2_, and O‴3-Na_1/3_NiO_2_ structures there are Ni sites with distinct oxidation states and Ni charge ordering in addition to Na vacancy ordering (Table 3). By contrast, in P″3-Na_1/2_NiO_2_, there is no observed charge disproportionation between the Ni sites, which are both approximately Ni^∼3.5+^. This is likely due to the crystallographic stripe arrangement of the Ni_(1)_ and Ni_(2)_ sites in the Ni layer of this phase, which cannot accommodate NiO_6_ environments with dramatically different sizes, without extreme octahedral distortion. The presence of Ni^*x*+^ charge ordering allows for local charge neutrality to be maintained in the vacancy ordered structures. The absence of Ni charge ordering in P″3-Na_1/2_NiO_2_ suggests that stable phase formation is driven by Na^+^/vacancy ordering, with flexibility in Ni^x+^ charge ordering facilitating charge neutrality within the framework of the most stable Na^+^/vacancy ordering. Further work is required to confirm this. We note that in most other Na_*x*_ *TM*O_2_ structures, the presence of Na^+^/vacancy ordering is accompanied by metal charge ordering, suggesting the two orderings are synergistic. When present, the two ordering schemes are arranged to ensure local charge neutrality *i*.*e*. occupied (vacant) Na sites, above/below oxidized (reduced) Ni sites. This is also favored from a space filling perspective as has been discussed in the P′3-Na_2/3_Cu_1/3_Mn_2/3_O_2_ phase,^44^ which has a similar charge and vacancy ordering scheme to P′3-Na_2/3_NiO_2_.^12^

Despite the oxidation of JT active Ni^3+^ to JT inactive Ni^4+^ in Na_*x*_NiO_2_, the structures retain a monoclinic distortion. We can parametrize the monoclinic distortion by decomposing it into in-plane and interplane components (see SI Section S-14), noting that the changes in stacking between the O and P phases limits meaningful comparison between these types of phases since the magnitudes of both distortion metrics are greater in the octahedral phases (squares in Figure S13). A significant decrease in the monoclinic distortion is observed on initial desodiation of O′3-NaNiO_2_ to P′3-Na_2/3_NiO_2_ but it then adopts smaller, yet finite, values for lower Na-contents. In O′3-NaNiO_2_ the monoclinic distortion is driven by the cooperative ordering of the JT active Ni^3+^ ions. This can be seen in the finite values obtained for the bond length distortion (BLDI), quadratic elongation and bond angle variance (BAV) (Table 3, SI Section S-11 Figure S10).

The van Vleck modes are another way of assessing octahedral distortions; unlike other metrics, these are uniquely sensitive to the symmetry of the JT distortion and hence can be used to decouple JT distortions from other octahedral distortions.^30^ The relevant modes are the Q_3_ and Q_2_ bond distortion modes describing Jahn–Teller elongation (2-long axial bonds, 4-short equatorial bonds and a 2-long, 2-medium, 2-short bond length distribution, respectively). JT distortions in NaNiO_2_ are purely Q_3_ whereas those in the JT d^4^ system LaMnO_3_ also have Q_2_ character. A phase space of possible JT-distorted configurations can be represented in a polar plot, mapped by magnitude (ρ_0_) $\left({\rho_{0}=\left(Q_{2}^{2}+Q_{3}^{2}\right)}^{1/2}\right)$ , and angle $\Phi =\mathrm{arc}\tan\left(\frac{Q_{2}}{Q_{3}}\right)$ . A polar plot showing the van Vleck distortion for each Ni site in the Na_*x*_NiO_2_ structures is shown in Figure 5. In general, the magnitude of the distortion (ρ_0_) decreases as the Ni oxidation increases from Ni^3+^ toward Ni^4+^. There is a less clear trend in the nature of the distortion (Φ) presumably reflecting how the distortions of the Ni sites accommodate the Na vacancy ordering. All of the octahedral phases (Figure 5, O′3 dark blue, O″3 purple, and O‴3 brown) retain archetypal JT elongations (Φ ≈ 0), while in the prismatic phases (Figure 5, P′3 orange, P″3 green) this is no longer the case, with NiO_6_ environments better described as JT compressions (Figure 5, orange circle and orange/green triangles). Even in the most highly oxidized samples with nominal Ni oxidation state Ni^3.90(4)+^, JT elongation distortions are still present. This is in contrast to other *ATM*O_2_ systems with no JT active ions (Figure 5, black hollow circles) where ρ_0_ is zero. The JT distortion present in NaNiO_2_ therefore persists in the structures adopted on desodiation even at high states of charge.

There are several Na_*x*_ *TM*O_2_ oxides that have JT active ions in one of the end members: d^4^ Mn^3+^ for *x* = 1, d^2^ Cr^4+^, and d^4^ Fe^4+^ for *x* = 0. For example, the JT distortion observed in NaMnO_2_ is much reduced in Na_0.437_MnO_2_, conversely JT distortions increase as Na is removed from Na_*x*_FeO_2_ and Na_*x*_CrO_2_. However, it is only in Na_*x*_NiO_2_ that the JT distortion is retained for all values of *x* (SI Section S-15, Figure S14). This suggests that the behavior of Na_*x*_NiO_2_ is distinct from other layered transition metal oxides and that there is some memory of the JT distortion down to the lowest sodium contents.

The work reported here has resolved the long-standing question regarding the structures of the Na_*x*_NiO_2_ phases formed on cycling. Unlike NaNiO_2_, the Li-containing analogue LiNiO_2_ does not usually exhibit cooperative JT ordering, which is often attributed to disorder.^48^ The structure of this phase has long been the subject of significant debate, because, even though it contains Ni^3+^, its average crystal structure has been reported to be rhombohedral (*R*3̅*m*),^10,55^ akin to non-JT analogue compounds such as NaCrO_2_ [Cr^3+^, d^3^], NaCoO_2_ [Co^3+^, d^6^], LiCoO_2_ [Co^3+^, d^6^], while JT distortions in the local structure have been predicted computationally,^34^ and observed experimentally *via* extended X-ray absorption fine structure spectroscopy (EXAFS).^56^ However, we recently reported that the average structure of defect-free LiNiO_2_ is indeed isostructural with NaNiO_2,_ with a monoclinic symmetry and a cooperative JT distortion. The previously observed rhombohedral symmetry is believed to arise from native antisite defects and off-stoichiometry in LiNiO_2_ synthesized *via* solid state methods, effectively “doping” the material with similarly sized Ni ions in the Li layer, and Li^+^ in the Ni layer.^7^ The voltage profile of defect-free LiNiO_2_ demonstrates a striking resemblance to that of NaNiO_2_ (Figure 1), exhibiting a range of step-like biphasic transitions, by comparison to the more sloping voltage profile reported for LiNiO_2_ containing antisite defects.^48^ This suggests that similar phase transitions driven by alkali metal-ion/vacancy ordering and transition metal charge ordering may play an underappreciated role in Li cathodes. Additionally, this link may explain why effective doping strategies (such as in nickel–manganese-cobalt [NMC] cathodes) modulate and control the phase transition behavior to a greater extent in Na-ion cathode materials than in Li-ion, where Li^+^ is smaller and more covalent than Na. Our herein reported insight into the structural evolution of Na_*x*_NiO_2_ may therefore inform understanding of structural evolution in LiNiO_2_.

While IE-LiNiO_2_ and NaNiO_2_ are isostructural, there is a significant difference in the magnitude of the JT distortion and the degree of monoclinic distortion. This may arise from the differences in ionic radii with the larger ionic radii of Na accommodating a larger JT distortion. A full investigation of this is beyond the scope of the present work but may also impact the phases formed on cycling defect-free IE-LiNiO_2_. The reported ordering phenomena, and their resultant influence on structural evolution in the electrochemistry of Na_*x*_NiO_2_, present a range of potential avenues of exploration in the development of next-generation Na-ion cathode materials. For example, through doping with multivalent cations in the Na-layer, a so-called “pillaring” effect has been observed to disrupt Na^+^/vacancy orderings.^32^ Further, targeted transition metal doping is widely used to enhance performance in Li^+^ and Na^+^ cathodes, such as the NMC series of cathodes, that were developed to minimize Co usage, while maintaining its beneficial effects on electrochemical cycling of Ni and Mn. While high Ni content cathodes have the beneficial effect of increasing accessible capacity and power, the ordering phenomena we report are highly detrimental to long-term performance. Targeted doping of aliovalent TM cations in the Ni layer has 2-fold potential to mitigate ordering, thus improve performance, both through disruption of the co-operative JT distortion,^32^ and disruption of the layer-gliding transition between O ↔ P phases.

## Conclusions

Using combined Rietveld refinement of structural models against SXRD and NPD data, we report complete structural solutions for the remaining three stable desodiated phases that form during the electrochemical desodiation of NaNiO_2_: P″3-Na_1/2_NiO_2_, O″3-Na_2/5_NiO_2_, and O‴3-Ni_1/3_NiO_2_. Each of these phases is observed to contain Na^+^/vacancy ordering in the Na-layer, and discrete Ni-charge ordering (as interpreted *via* BVS analysis) is observed in O″3-Na_2/5_NiO_2_ and O‴3-Na_1/3_NiO_2_. The superstructures that form are predominantly stabilized by the minimization of electrostatic Na^+^-Na^+^ repulsions in the Na layer. Even in the most oxidized structures, finite JT distortions are observed. Sequential Rietveld refinement of SXRD data provided evidence of a transient P‴3-Na_1/2<*x*<2/3_NiO_2_ phase, which was previously not isolable *ex situ*, along with confirming a solid-solution phase transition behavior between the O″3-Na_2/5_NiO_2_ and O‴3-Na_1/3_NiO_2_ phases at the top of charge.

Building on the structural evolution established by this work, structure–property relationships may now be established through investigations of Na^+^-mobility and dynamics in NaNiO_2_, facilitating greater understanding of the de/sodiation mechanisms. Further, based on the atomistic structures presented, it is now possible to consider targeted doping strategies to mitigate the reported ordering phenomena in Na_*x*_NiO_2_, facilitating rational design of longer lasting Na-ion battery cathodes. Our work provides a platform to understand structure and dynamics in Ni-rich layered cathodes, including both Li^+^- and Na^+^-containing cathodes including defect-free LiNiO_2_,^48^ and NMC-type layered alkali transition metal oxides.

## Supplementary Material





## Notes

A schematic of the *operando* SXRD cell, XANES data, cell sample masses, a description of the ToF Stephens’-type anisotropic peak broadening macro (which has itself been provided with the manuscript submission), plots of SXRD data for all phases, details of the combined refinement structures of each phase, information on refinements pertaining to transient phases, refinement on *operando* SXRD data for the O‴3-Na_1/3_NiO_2_ phase, expanded Na-layer schematics for each phase, additional plots of distortion parameters as a function of magnitude of octahedral distortion, refinements on data containing the “layer-collapse” O1 phase, a plot of the change in NaO_6_ environments on desodiation, a description of the herein used quantification metrics for monoclinicity, and a comprehensive comparison plot describing the evolution of octahedral distortion across the first row A_*x*_ *TM*O_2_ phases (PDF)
Topas macro and template for Stephens’-type anisotropic peak broadening of time-of-flight neutron powder diffraction data (ZIP)

## ∇

Stanford Synchrotron Radiation Lightsource, SLAC National Accelerator Laboratory, Menlo Park, California 94025, United States
