# Supplementary material for: Evolution of Charge and Orbital Ordering, and Cation Vacancy Ordering During Electrochemical Desodiation of Na x NiO2
Source: J Am Chem Soc. 2026 May 20;148(21):22062–76. doi: 10.1021/jacs.6c03074 (PMC13244472; doi:10.1021/jacs.6c03074)
Supplement: Supplementary file 1 [file ja6c03074_si_001.pdf]

Supplementary Information: Evolution of Charge and Orbital Ordering, and Cation Vacancies During Electrochemical Desodiation of Na_xNiO_2

James M. A. Steele,[1, 2] Joshua D. Bocarsly,[3] Liam A. V. Nagle-Cocco,[2,4] George S. Phillips,[1] Farheen N. Sayed,[1] Giulio I. Lampronti,[5] Fabio Orlandi,[6] Pascal Manuel,[6] Iuliia Mikulska,[7] Clare P. Grey [1] & Siân E. Dutton* [2]*

[1] Yusuf Hamied Department of Chemistry, University of Cambridge, Cambridge, CB2 1EW, UK

[2] Cavendish Laboratory, University of Cambridge, JJ Thomson Avenue, Cambridge, CB3 0US, UK

[3] Department of Chemistry and Texas Center for Superconductivity, University of Houston, Houston, TX 77004, USA

[4] Current address: Stanford Synchrotron Radiation Lightsource, SLAC National Accelerator Laboratory, Menlo Park, CA 94025, USA

[5] Department of Materials Science and Metallurgy, University of Cambridge, Cambridge CB3 0FS, UK

[6] ISIS Neutron and Muon Source, Rutherford Appleton Laboratory, STFC, UKRI, Harwell Science and Innovation Campus, Didcot OX11 0QX, UK

[7] Diamond Light Source, Harwell Science and Innovation Campus, Didcot OX11 0DE, UK

[*] Email: cpg27@cam.ac.uk, sed33@cam.ac.uk

Contents

S-1: *Operando* Synchrotron X-ray Diffraction Cell 3
S-2: X-ray Absorption Near-Edge Structure 4
S-3: Swagelok Cell Sample Masses 6
S-4: TOPAS Macro for Stephens'-Type Anisotropic Peak Broadening of Time-of-Flight Neutron Powder Diffraction Data 7
S-5: SXRD Data 8
S-6: Details of the Combined Refinement Structures 9
S-7: Rietveld Refinement of Data Containing the Non-Isolable P'''3-$Na_{1/2<x<2/3}NiO_2$ Phase 11
S-8: Rietveld Refinement of Data Containing the Non-Isolable O''3δ- $Na_{1/3<x<2/5}NiO_2$ Phase 14
S-9: Refinement of O'''3-$Na_{1/3}NiO_2$ Model Against *Operando* SXRD Only 15
S-10: Expanded Na Layer Na^+/Vacancy Ordering Schematics 16
S-11: Distortion Parameters as a Function of Octahedral Distortion Magnitude (ρ_0) 17
S-12: High Voltage O1-$Na_{0<x<1/3}NiO_2$ Layer Collapse Phase 18
S-13: NaO_6 Environments as a Function of Desodiation 19
S-14: Quantifying Monoclinicity in Na_xNiO_2 vs Li Analogues 20
S-15: Evolution of Octahedral Distortion in First Row A_xTMO_2 Compounds 22

S-1: *Operando* Synchrotron X-ray Diffraction Cell

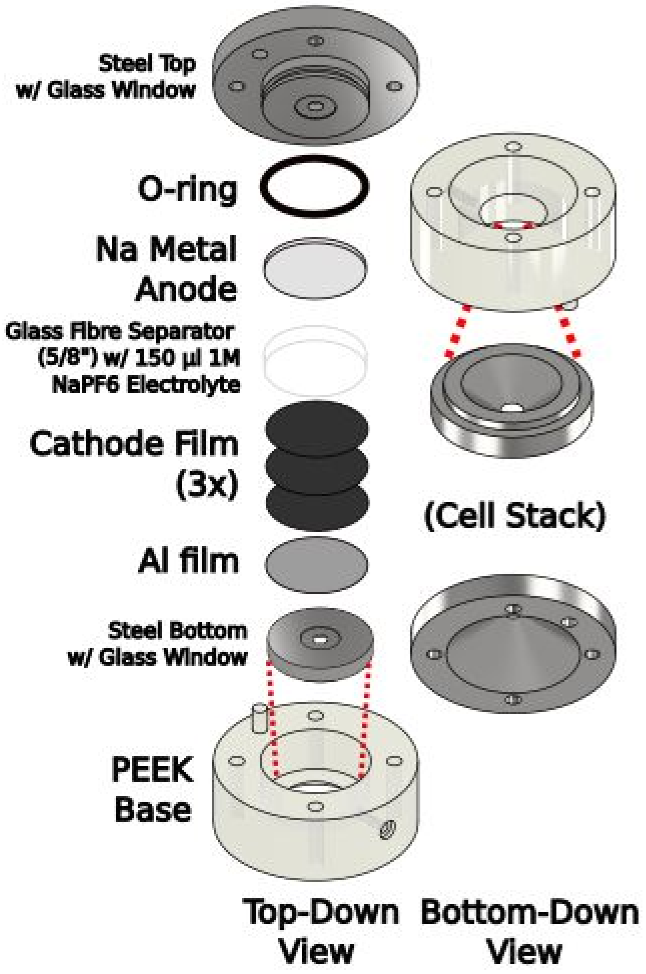

Figure S1: Diagram of the operando SXRD cell and 3-cathode cell stack.

S-2: X-ray Absorption Near-Edge Structure

X-ray absorption spectroscopy (XAS) measurements were obtained at the Ni K-edge for *ex situ* desodiated samples, and the pristine O'3-$NaNiO_2$ material (Figure S2). Edge energy was estimated *via* fitting of a Gaussian peak to the first derivative of absorption with respect to measured energy (Figure S3), in energy range 8336 - 8353 eV, taking the energy of the maximum to be the Ni K-edge energy. This was observed to shift to higher energies with decreasing Na concentration.[11] This shift is consistent with progressive oxidation of Ni^{3+} as sodium is removed from Na_xNiO_2.

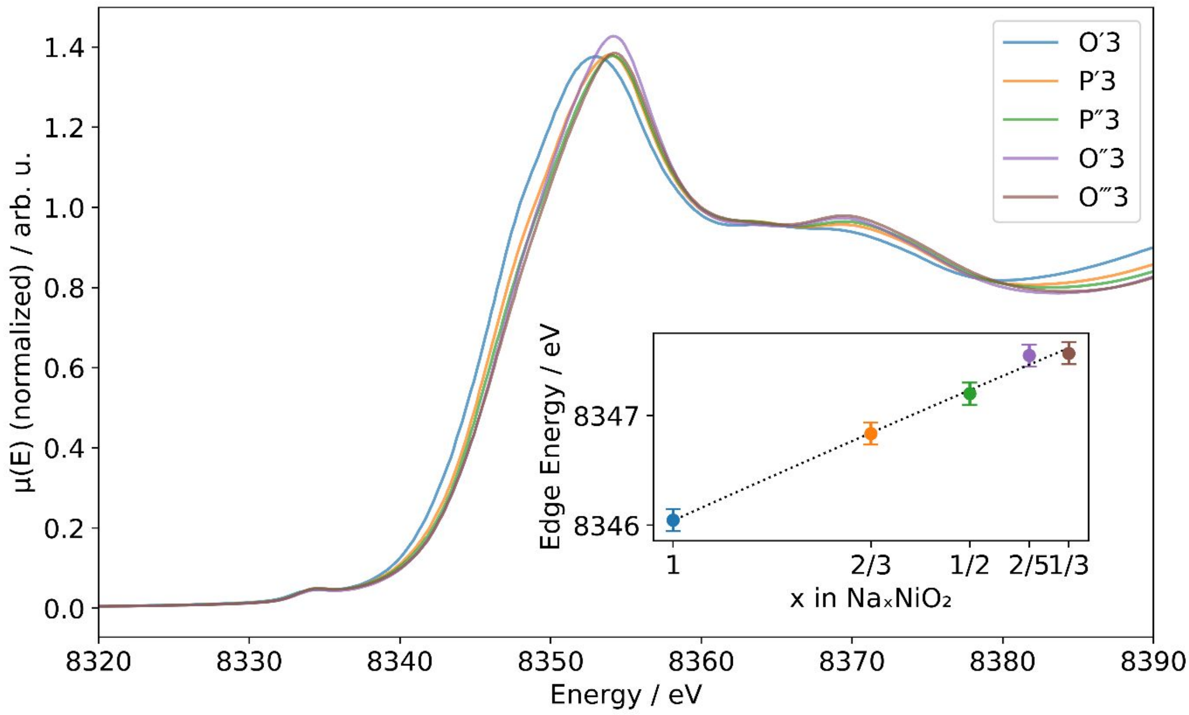

Figure S2: Ni K-edge XAS spectra collected for each charge phase. The inset shows edge energy as a function of Na content, with the black dotted line included as a visual guide.

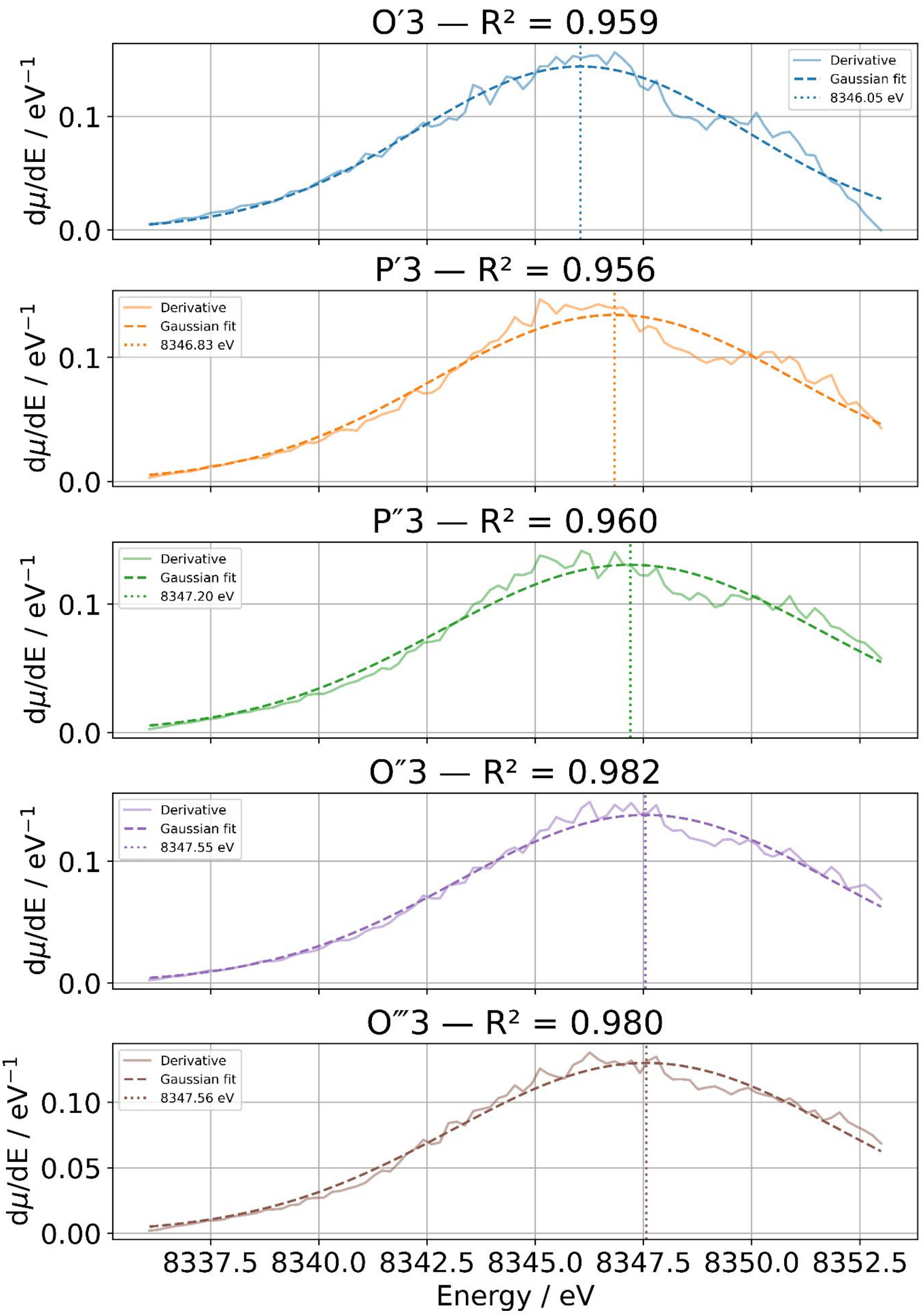

Figure S3: Gaussian fitting of the first derivative of Ni K-edge XAS spectra collected for each charge phase in energy range 8336 - 8353 eV. The energy at the peak of the first derivative is taken to be the Ni K-edge energy.

S-3: Swagelok Cell Sample Masses

Table S1: Sample masses, and calculated ratios for the ex situ samples reported in this work.

Sample Identity	Mass Active Cathode /mg	Mass Conductive Carbon /mg	Total Mass /mg	Calculated Ratio Cathode:Carbon
P''3-$Na_{1/2}NiO_2$	98.33	41.97	140.30	70:30
O''3-$Na_{2/5}NiO_2$	100.78	43.02	143.80	70:30
O'''3-$Na_{1/3}NiO_2$	99.80	40.60	140.40	71:29

S-4: TOPAS Macro for Stephens'-Type Anisotropic Peak Broadening of Time-of-Flight Neutron Powder Diffraction Data

Macros were used to model Stephens'-type anisotropic peak broadening of the TOF NPD data presented in this work. To facilitate usage of these macros in the work of interested users, two files have been included in the supplementary information of this paper. "tof_stephens_peak_types.inc" contains all of the required macros, and may be included in a user's TOPAS installation *via* the instructions contained in the file. "tof_stephens_peak-types-template.inp" contains templates for the callable sections of the macros for each crystal class, to be included in a user's TOPAS input file.

S-5: SXRD Data

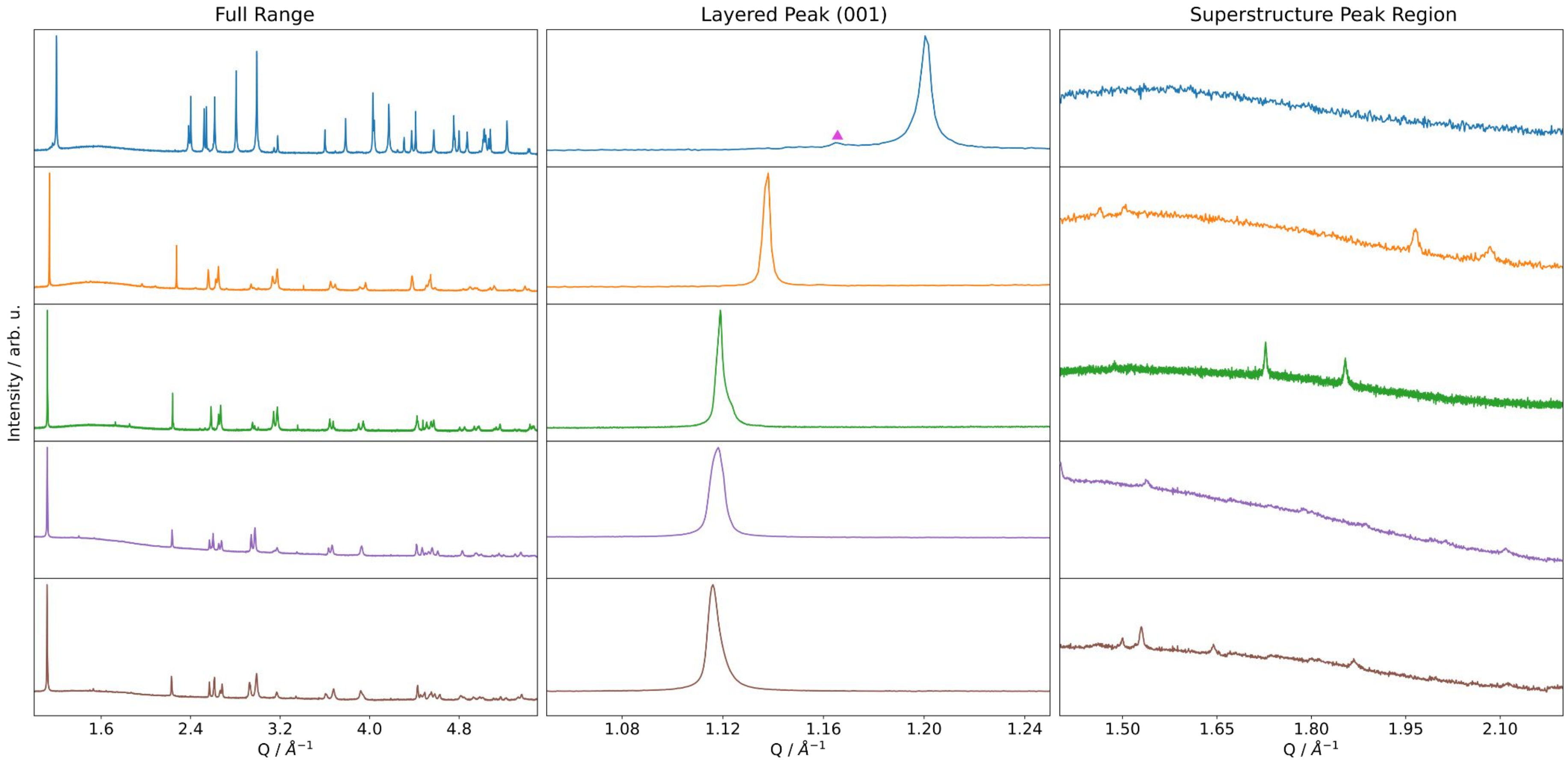

Figure S4: SXRD patterns of O'3-$NaNiO_2$ cathode powder (blue), and ex situ samples P'3-$Na_{2/3}NiO_2$ (orange), P''3-$Na_{1/2}NiO_2$ (green), O''3-$Na_{2/5}NiO_2$ (purple), and O'''3-$Na_{1/3}NiO_2$ (brown). A minor O''''3-$Na_{2/3<x<1}NiO_2$ impurity in the O'3-$NaNiO_2$ sample was identified (pink triangle). For visual clarity three ranges are plotted: wide Q-range 1.0 – 5.5 Å (left), layered (001) peak 1.05 – 1.25 Å (centre), and superstructure peaks 1.4 – 2.2 Å (right). Note patterns for O'3-$NaNiO_2$ P'3-$Na_{2/3}NiO_2$, and P''3-$Na_{1/2}NiO_2$ were collected using multi-analyser crystal detectors, whilst O''3-$Na_{2/5}NiO_2$ and O'''3-$Na_{1/3}NiO_2$ were collected using a position sensitive detector.

S-6: Details of the Combined Refinement Structures

Table S2: Structural parameters for the P″3-$Na_{1/2}NiO_2$ structure as determined from combined Rietveld refinement of SXRD and NPD data (R_{wp} = 1.901 %, χ^2 = 1.111). Unrefined (special) positions given in italic.

**Space group**	**a (Å)**	**b (Å)**	**c (Å)**	**β (°)**	**Unit-cell volume (Å³)**	**Unit-cell volume per f.u. (Å³)**
P2₁/m	4.91947(9)	5.66324(11)	5.82204(15)	105.63782(4)	156.199(8)	39.138(2)
**Element**	**Wyckoff position**	**x**	**y**	**z**	**Occupancy**	**B_{iso} (Å²)**
$Ni_{(1)}$	*2a*	*0*	*0*	*0*	1	1.22(25)
$Ni_{(2)}$	*2e*	0.508(3)	*0.25*	0.011(2)	1	1.33(27)
$Na_{(1)}$	*2e*	0.299(4)	*0.25*	0.494(3)	1	3.24(34)
$O_{(1)}$	*4f*	0.399(3)	0.004(5)	0.188(3)	1	1.29(40)
$O_{(2)}$	*2e*	0.110(6)	*0.25*	0.819(5)	1	0.75(45)
$O_{(3)}$	*2e*	0.881(6)	*0.25*	0.158(4)	1	1.23(49)

*Table S*3: Structural parameters for the O″3-$Na_{2/5}NiO_2$ structure as determined from combined Rietveld refinement of SXRD and NPD data (R_{wp} = 1.230 %, χ^2 = 2.829). Unrefined (special) positions given in italic.

**Space group**	**a (Å)**	**b (Å)**	**c (Å)**	**β (°)**	**Unit-cell volume (Å³)**	**Unit-cell volume per f.u. (Å³)**
C2/m	4.94085(4)	14.03925(13)	5.86783(4)	106.51831(10)	390.228(40)	39.023(4)
**Element**	**Wyckoff position**	**x**	**y**	**z**	**Occupancy**	**B_{iso} (Å²)**
$Ni_{(1)}$	*2a*	*0*	*0*	*0*	1	2.08(14)
$Ni_{(2)}$	*4g*	*0*	0.1990(3)	*0*	1	1.96(8)
$Ni_{(3)}$	*4g*	*0*	0.3996(4)	*0*	1	1.34(5)
$Na_{(1)}$	*4h*	*0*	0.1114(5)	*0.5*	1	3.63(9)
$O_{(1)}$	*4i*	0.270(2)	*0*	0.8096(17)	1	1.12(25)
$O_{(2)}$	*8j*	0.2877(16)	0.2006(8)	0.8443(8)	1	3.06(13)
$O_{(3)}$	*8j*	0.2799(12)	0.4009(8)	0.8183(11)	1	1.36(16)

*Table S*4: Structural parameters for the O′′′3-$Na_{1/3}NiO_2$ structure as determined from combined Rietveld refinement of SXRD and NPD data (R_{wp} = 2.175 %, χ^2 = 5.652). Unrefined (special) positions given in italic.

**Space group**	**a (Å)**	**b (Å)**	**c (Å)**	**β (°)**	**Unit-cell volume (Å^3)**	**Unit-cell volume per f.u. (Å^3)**
C2/m	4.93408(11)	8.38899(18)	5.90396(12)	107.142(3)	233.520(9)	38.920(2)
**Element**	**Wyckoff position**	**x**	**y**	**z**	**Occupancy**	**B_{iso} (Å^2)**
$Ni_{(1)}$	2*a*	*0*	0	0	1	1.69(20)
$Ni_{(2)}$	4*g*	*0.5*	0.1663(5)	0	1	1.12(9)
$Na_{(1)}$	2*d*	*0.5*	0	0.5	1	0.21(19)
$O_{(1)}$	4*i*	0.7313(13)	0	0.1918(6)	1	1.17(24)
$O_{(2)}$	8*j*	0.2854(7)	0.3246(6)	0.8365(4)	1	1.81(14)

S-7: Rietveld Refinement of Data Containing the Non-Isolable P'''3-$Na_{1/2<x<2/3}NiO_2$ Phase

Figure S5 shows a slice of the *operando* SXRD data, taken at the point at which the greatest wt% of the transient P'''3-$Na_{1/2<x<2/3}NiO_2$ phase was present (47.7[2] wt%), two Rietveld refinements, and a heatmap of the SXRD slices in the region of the P'3-$Na_{2/3}NiO_2$ -> P'''3-$Na_{1/2<x<2/3}NiO_2$ -> P''3-$Na_{1/2}NiO_2$ transition. The top plot is a refinement which contains only P'3-$Na_{2/3}NiO_2$ and P''3-$Na_{1/2}NiO_2$ structures, and is poor fit (R_{wp} = 8.423 %) with a distinct layered (001) peak between their own. The lower plot shows a Rietveld refinement in which a third phase, an approximation of the intermediate phase (the P''3 structure with its own lattice parameters) was included. This represents a much better fit (R_{wp} = 4.640 %).

Figure S6 shows 3 SXRD patterns overlaid, the P'3-$Na_{2/3}NiO_2$ phase only (orange), P''3-$Na_{1/2}NiO_2$ phase only (green), and the slice which presents the greatest weight percent of the transient P'''3-$Na_{1/2<x<2/3}NiO_2$ phase (red). Two regions of interest are presented, enhanced for visual clarity. The left plot shows the superstructure peak region, the right plot is a region of peaks at higher Q. Both P'3-$Na_{2/3}NiO_2$ and P''3-$Na_{1/2}NiO_2$ have distinct superstructure peaks. In the red XRD pattern, there is still some residual intensity from the P'3-$Na_{2/3}NiO_2$ peaks (~1.46/1.50 Å), no intensity from the P'''3-$Na_{1/2<x<2/3}NiO_2$ peaks (~1.72/1.85 Å), and its own unique, broad superstructure peak (~1.78 Å). We hypothesise that this could arise due to a $Na_{4/7}NiO_2$ superstructure, but it has not been possible at this time to index this. Attempts to fit this superstructure peak were hampered by the fact that the P'''3-$Na_{1/2<x<2/3}NiO_2$ phase does not exist in isolation.

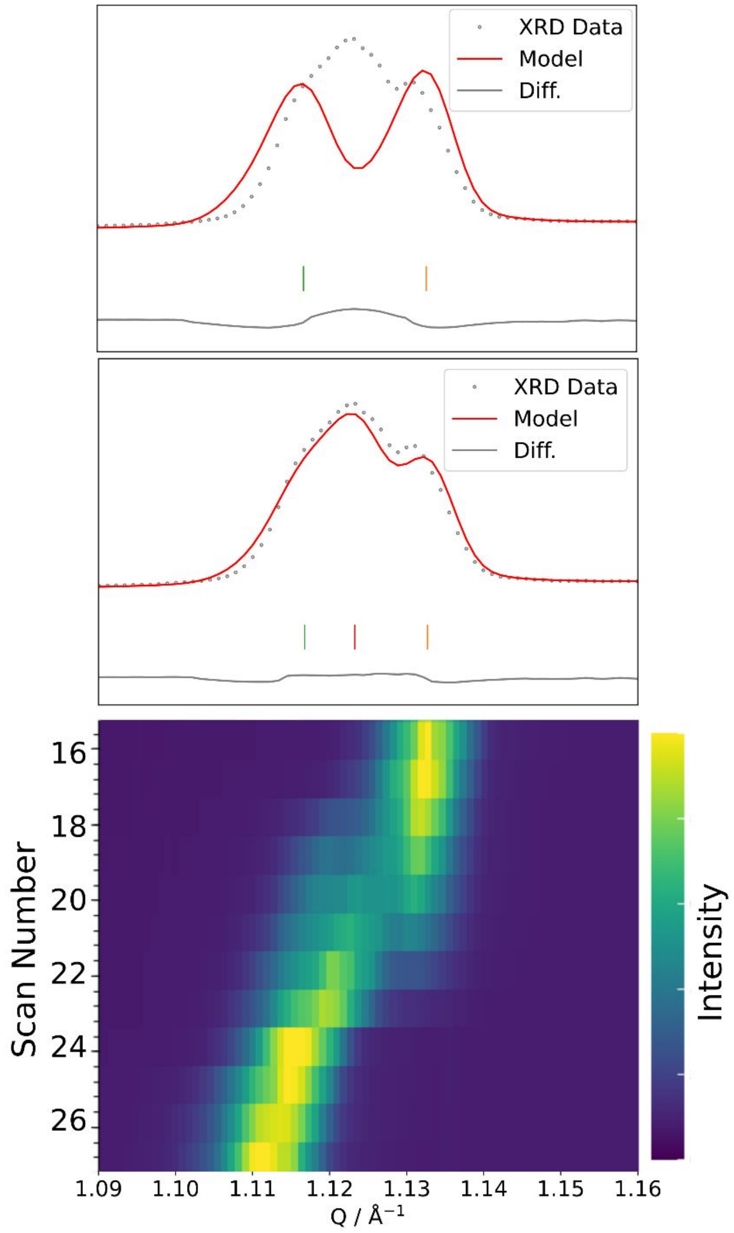

Figure S5: Refinements and heatmap of operando SXRD data (grey circles), models (red lines), differences (grey lines). Tick marks are displayed below for each phase: P'3-$Na_{2/3}NiO_2$ (orange), P''3-$Na_{1/2}NiO_2$ (green), and approximation of the transient P'''3-$Na_{1/2<x<2/3}NiO_2$ phase (red). Top refinement does not contain the transient phase (R_{wp} = 8.423 %), bottom plot contains the transient phase (R_{wp} = 4.640 %). The bottom image is a heatmap, focusing on the layered (001) peak region across the transition, demonstrating the 3 unique phases present. Heatmap y-axis top-bottom is onset of prismatic phase transition (orange lines in Figure S6) – end of prismatic phase transition (green lines in Figure S6).

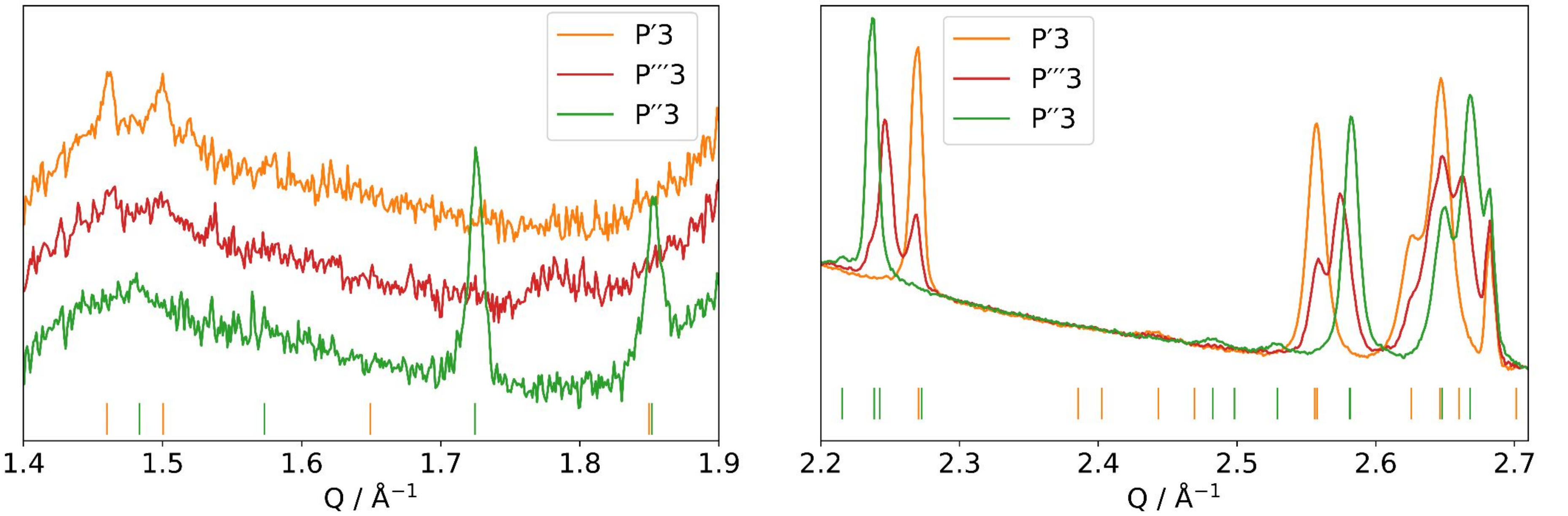

Figure S6: Operando SXRD data for each phase: P'3-$Na_{2/3}NiO_2$ (orange), P''3-$Na_{1/2}NiO_2$ (green), and most prominent example of the transient P'''3-$Na_{1/2<x<2/3}NiO_2$ phase (red). Tick marks are displayed below for the two well defined structural models. Two regions are presented for visual clarity: superstructure peak region (left), and a higher Q region with sufficient resolution and separation to demonstrate distinct structural differences between each phase (right). The red diffraction pattern contains some residual intensity from the other phases.

S-8: Rietveld Refinement of Data Containing the Non-Isolable O''3δ- $Na_{1/3<x<2/5}NiO_2$ Phase

Figure S7 displays SXRD data from the region of the O''3-$Na_{2/5}NiO_2$ -> O''3δ-$Na_{1/3<x<2/5}NiO_2$ -> O'''3-$Na_{1/3}NiO_2$ transition, displaying smooth shifting of peaks in the layered peak (001) (a) and higher Q region (c). The overlaid data in the superstructure peak region (b, top) demonstrates the lack of additional superstructure peaks intensity in scans between the loss of O''3-$Na_{2/5}NiO_2$, and emergence of O'''3-$Na_{1/3}NiO_2$. Whilst the waterfall plot of all scans in this region (b, bottom) may hint at some new broad feature at ~1.45 Å, the overlaid plot (b, top) establishes that this is residual intensity from the other phases, or background attributable to formation of side products with low crystallinity, likely resultant from electrolyte degradation due to high voltages at the top of charge.

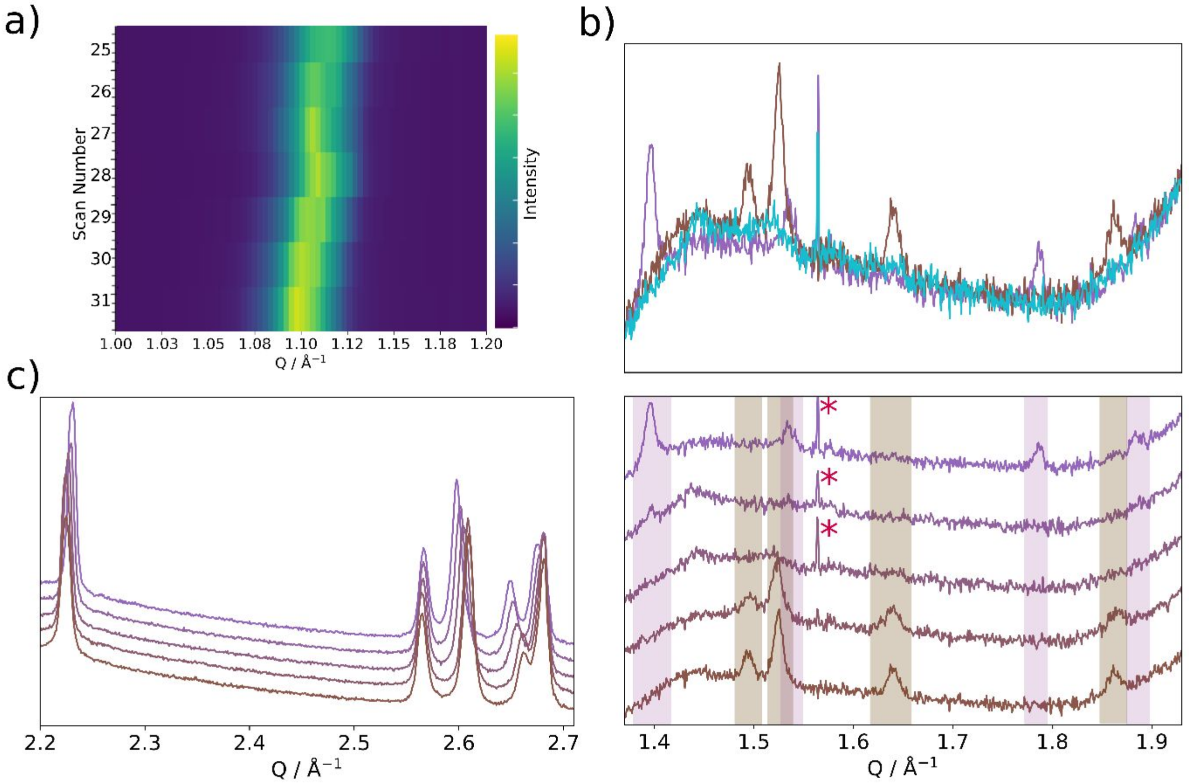

*Figure S7a-c: Operando SXRD data for the octahedral transition at the top of charge. Y-axis top-bottom in all plots and heatmap is onset of octahedral phase transition (purple lines in plots) – end of charge (brown lines in plots) a) heatmap of the layered peak (001) reflection region. b) overlaid (top) and waterfall (bottom) data within the superstructure peak region; a sharp spurious peak due to clipping of the steel cell body by the x-ray beam in these scans is identified by a red *. c) Higher Q region demonstrating continuous shifting of peaks across the scans.*

S-9: Refinement of O'''3-$Na_{1/3}NiO_2$ Model Against *Operando* SXRD Only

As noted in the main text, the model produced by ISODISTORT and refined against the *ex situ* SXRD and NPD data resulted in some of the predicted superstructure peak intensity arising in the calculated diffraction patterns, which were not present in the experimentally observed data. However, on inspection of the *operando* SXRD, fitting with the same model resultant from the *ex situ* combined refinement, demonstrated that the missing superstructure peak intensity from the *ex situ* data, was in fact present in the *operando SXRD* data, suggesting that the missing reflections exist for only a short time-/Na content-window in the electrochemistry. Alternatively, this could be due to relaxation, or reaction of the *ex situ* samples between sample preparation and data collection. As this is the sample with the least Na, and therefore widest interlayer distance, it is not unreasonable that this sample would be the most susceptible to reaction with trace amounts of atmospheric contaminants, such as moisture.

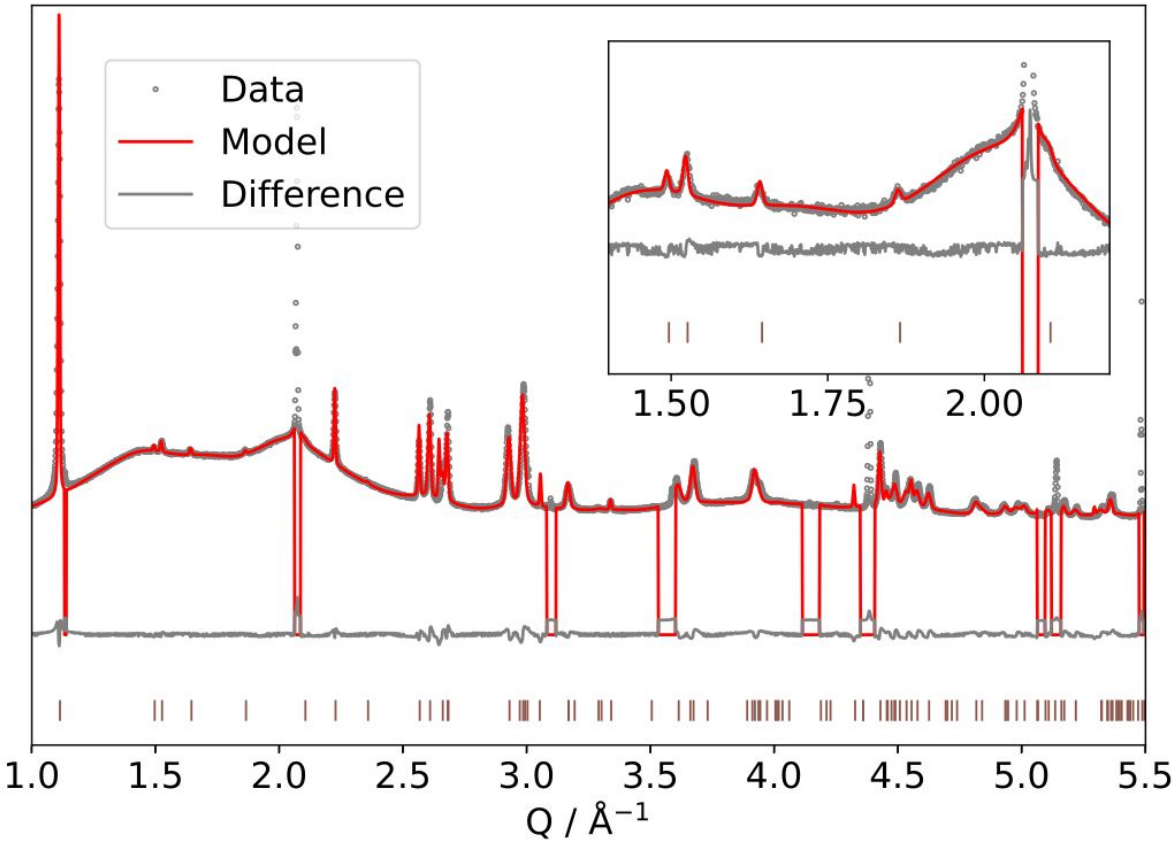

Figure S8: Fit of the combined refinement model (red line) to operando SXRD data of O'''3-$Na_{1/3}NiO_2$. R_{wp} / χ^2 = 8.232 % / 5.993 . Data collected at room temperature (approximately 25 °C). Square root of intensity is plotted on the y-axis for visual clarity. Inset shows the superstructure peak region. The drops in the model and difference represent excluded regions where Steel, Na or Al peaks from the cell are present, which it is not possible to fit due to strong preferential orientation in the rolled metals.

S-10: Expanded Na Layer Na+/Vacancy Ordering Schematics

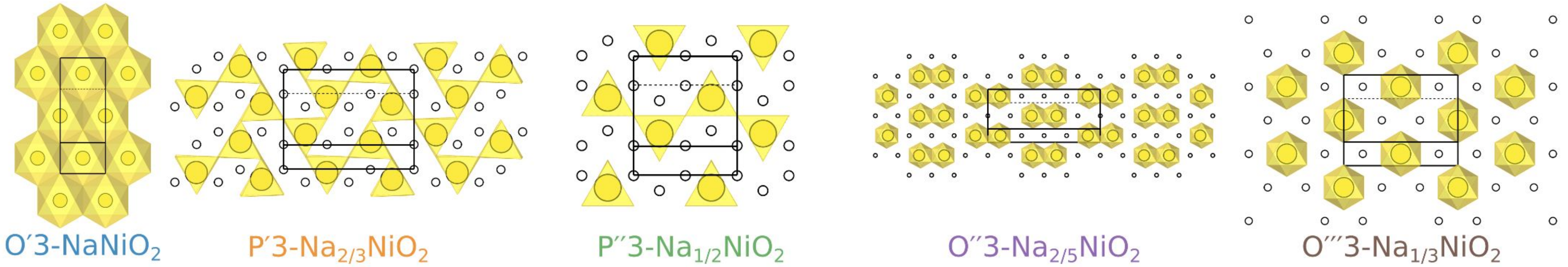

Figure S9: Na^+/vacancy ordering in the Na layer. Expansions of the unit cells of each structure to illustrate connectivity and nomenclature, left to right: no vacancies, zigzag, chains, pairs, isolated. Corresponding to 6 -> 3 -> 2 -> 1 -> 0 edge-sharing interactions respectively.

S-11: Distortion Parameters as a Function of Octahedral Distortion Magnitude (ρ_0)

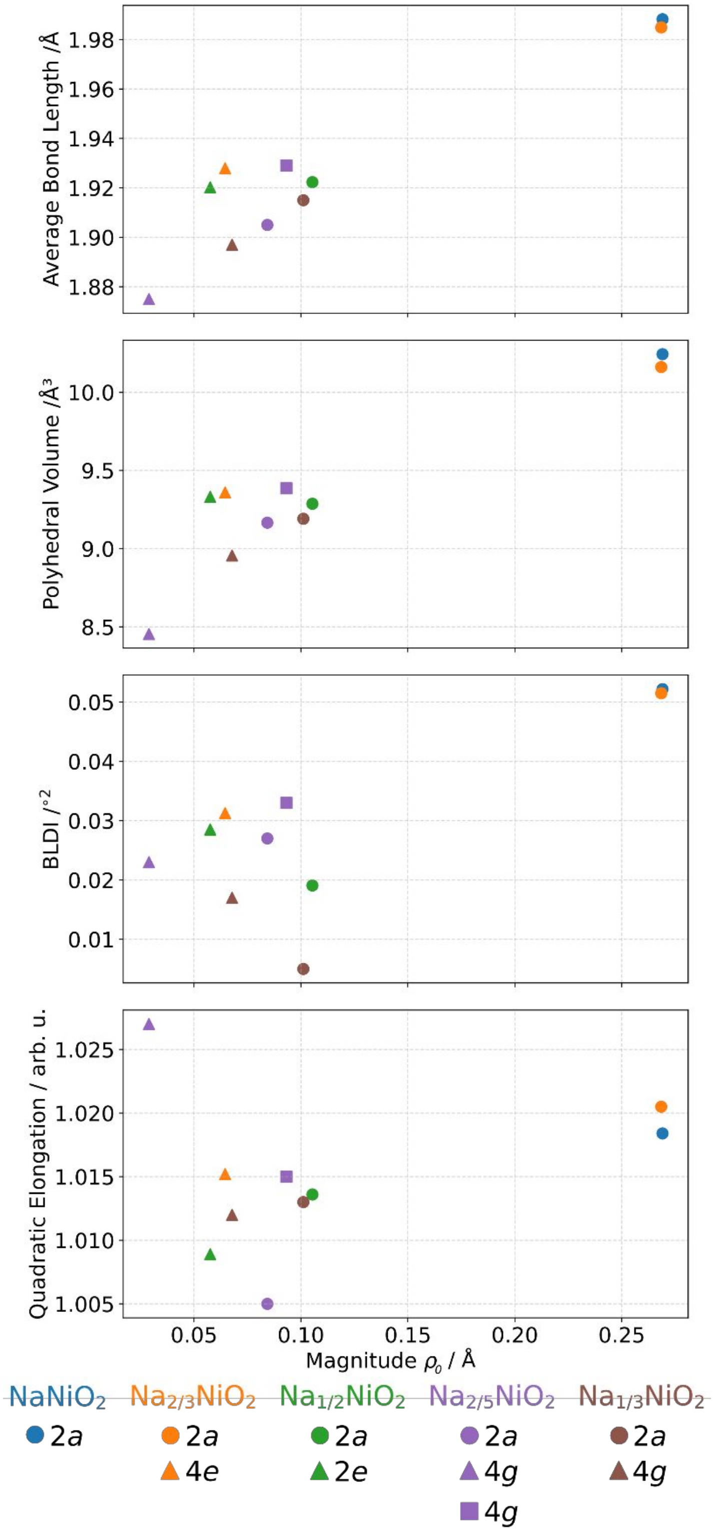

Figure S10: Characteristics of the octahedra present in the desodiated phases, as a function of magnitude of octahedral distortion ρ_0. Marker colours denote desodiated phases O'3-$NaNiO_2$ (light blue), P'3-$Na_{2/3}NiO_2$ (orange), P''3-$Na_{1/2}NiO_2$ (green), O''3-$Na_{2/5}NiO_2$ (purple), O'''3-$Ni_{1/3}NiO_2$ (brown).Shapes denote crystallographic sites $Na_{(1)}$ (circles), $Na_{(2A)}$ (triangles), and $Na_{(2B)}$ (square, note there is only one such site and it overlaps with the $Ni_{(1)}$ site of the same phase).

S-12: High Voltage O1-$Na_{0<x<1/3}NiO_2$ Layer Collapse Phase

One of the attempts to make a phase pure *ex situ* sample of O'''3-$Na_{1/3}NiO_2$, involved holding a cell at 4.5 V for 48 h. This resulted in significant intensity of a new layered (001) peak at much higher Q than any previously observed, commensurate with the emergence of a phase with significantly smaller interlayer spacing. Rietveld refinement on the highest resolution NPD bank (which still covered sufficient range to include the O'''3-$Na_{1/3}NiO_2$ layered peak, bank 4), resulted in a fit containing majority ~82-83 wt % O'''3-$Na_{1/3}NiO_2$, ~17 wt % O1-$Na_{0<x<1/3}NiO_2$, and trace amounts of unreacted (i.e. electrochemically disconnected) O'3-$NaNiO_2$. Our model for the O1-$Na_{0<x<1/3}NiO_2$ phase was based on the structure of the layer collapse phase in the electrochemistry of O3-$LiCoO_2$ reported by Tarascon *et al.,* and termed "CoO_2".[40] The lattice parameters were allowed to vary (reflective of the size difference between Na^+ and Li^+). By comparison to the parent O'3-$NaNiO_2$ phase, the fit O1-$Na_{0<x<1/3}NiO_2$ "NiO_2" phase had approximately 30 % contraction in its interlayer spacing (3.66[50] Å vs 5.23 Å), commensurate with the description of the O1-$Na_{0<x<1/3}NiO_2$ phase reported in $NaNi_{0.9}Ti_{0.1}O_2$ by An *et al.*.[31]

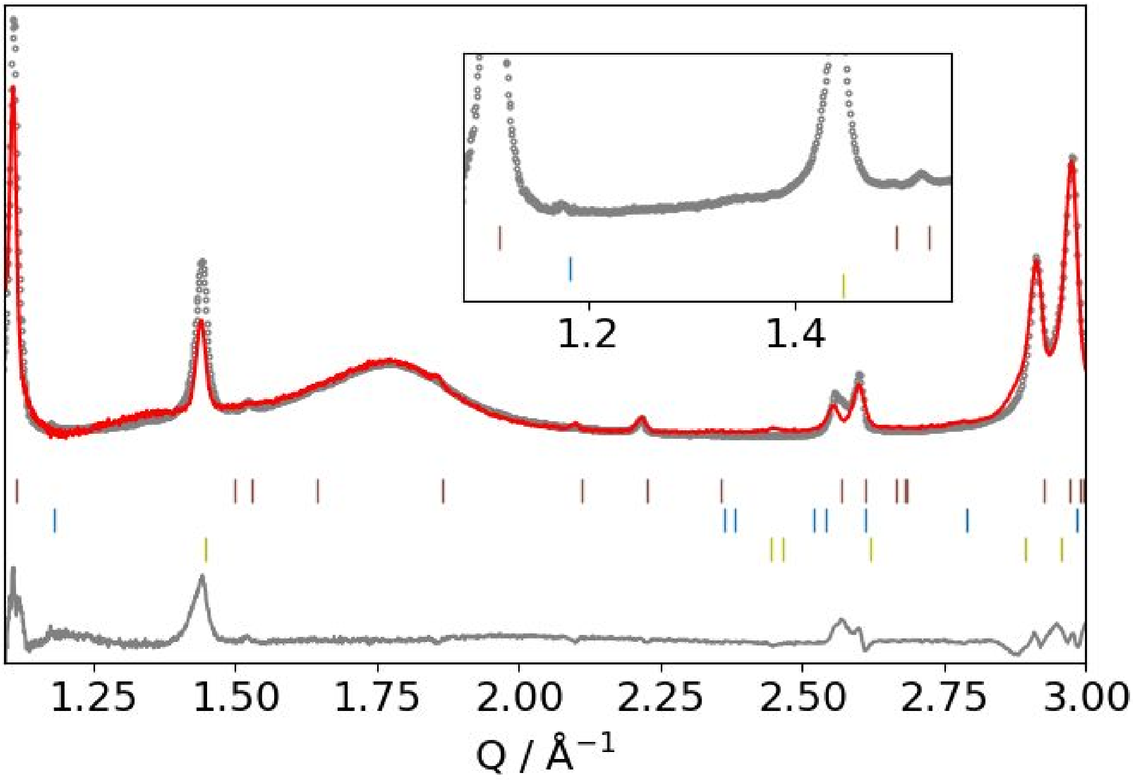

Figure S11: Refinement against NPD (average 2θ of bank pairs 121.66 °), data (grey circles), model (red line), difference (grey line). Tick marks are displayed below for each phase: O'''3-$Na_{1/3}NiO_2$ (brown), O'3-Na_1NiO_2 (blue), and layer collapse "~NiO_2" O1-$Na_{0<x<1/3}NiO_2$ (yellow). R_{wp} = 6.033 %. Data collected at room temperature (approximately 25 °C). Inset presents region in which layered peaks, and tick marks for the phases present, are present for visual clarity.

S-13: NaO_6 Environments as a Function of Desodiation

It is observed that, whilst the NiO_6 octahedra contract as Ni^{x+} is oxidised on sodium removal, the NaO_6 environments expand, with increasing bond lengths (Figure S12). This is reflective of decreased Na^+-Na^+ repulsive electrostatic interactions as the amount of edge-sharing occupied Na sites is decreased. The volumes of the octahedral and prismatic Na sites are not directly comparable, leading to the apparent contraction in the volume plot. Nonetheless, it is clear from the increasing bond lengths that the overall trend is elongation of Na-O bond lengths with decreasing Na content.

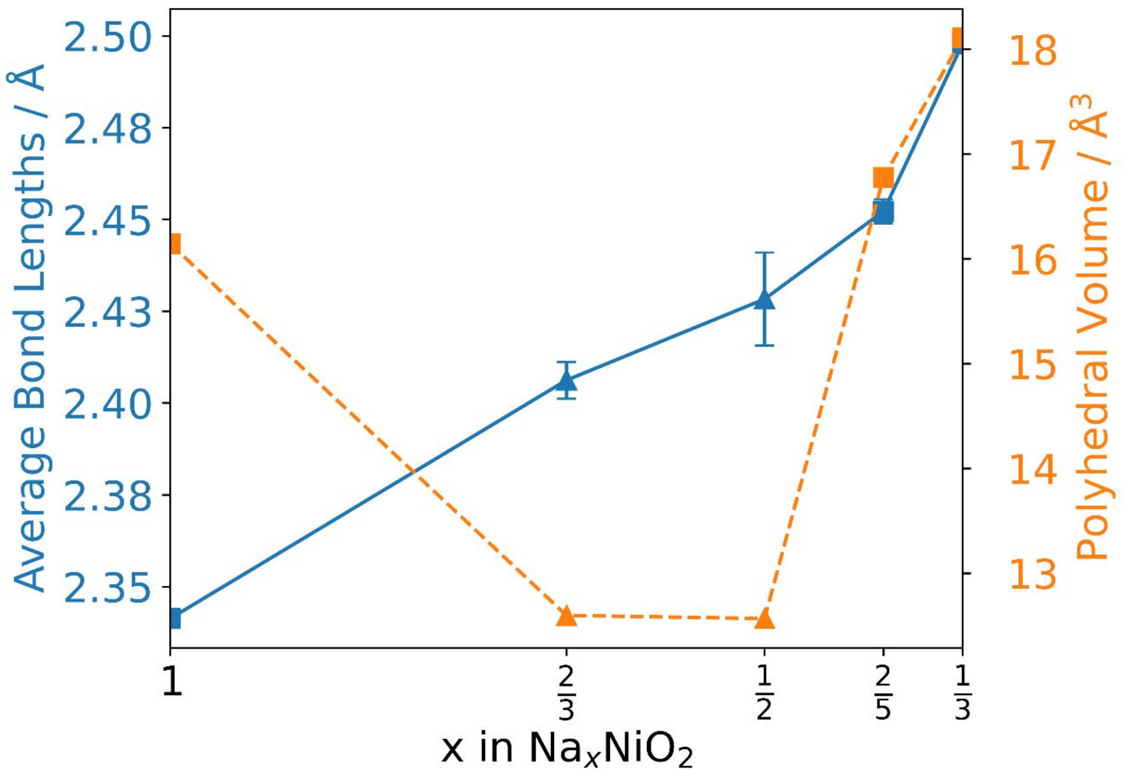

Figure S12: Average Na-O bond lengths (blue) and polyhedral volumes (orange) for the NaO_6 octahedra (squares) and prisms (triangles) present in the Na_xNiO_2 phases.

S-14: Quantifying Monoclinicity in Na_xNiO_2 vs Li Analogues

Degrees of monoclinicity can be quantified *via* two orthogonal modes which break the ideal rhombohedral symmetry. The "in-plane" distortion quantified *via* the a_{mon}/b_{mon} ratio (where a value of $\sqrt{3}$ corresponds to no monoclinic distortion), whilst the "inter-plane" layer shearing is quantified *via* the delta angle (where a value of 90˚ corresponds to no monoclinic distortion). These metrics are defined in the below equations SE1/SE2.

$$In-plane\ Distortion = \frac{a_{mon}}{b_{mon}} \quad \text{(SE1)}$$

$$Inter-plane\ Distortion = \delta \approx \beta - \arcsin\left(\frac{a_{mon}}{3c_{mon}}\right) \quad \text{(SE2)}$$

These distortion metrics were calculated for each Na_xNiO_2 and the benchmark IE-LNO/LNO phases. The ideal O3 rhombohedral structure demonstrated in LNO is denoted by the black dotted line in Figure S14, with deviation from this representing monoclinicity in the structure. The magnitude of inter-plane monoclinicity was greater than IE-LNO and LNO for all Na_xNiO_2 phases, whilst the in-plane monoclinicity was greater than IE-LNO for all octahedral phases. It is less possible to make direct comparison of monoclinicity by these metrics as defined by Phillips *et al.,*[46] in the context of the prismatic phases, as demonstrated by the interplane monoclinicity angle <90 º unique to these structures.

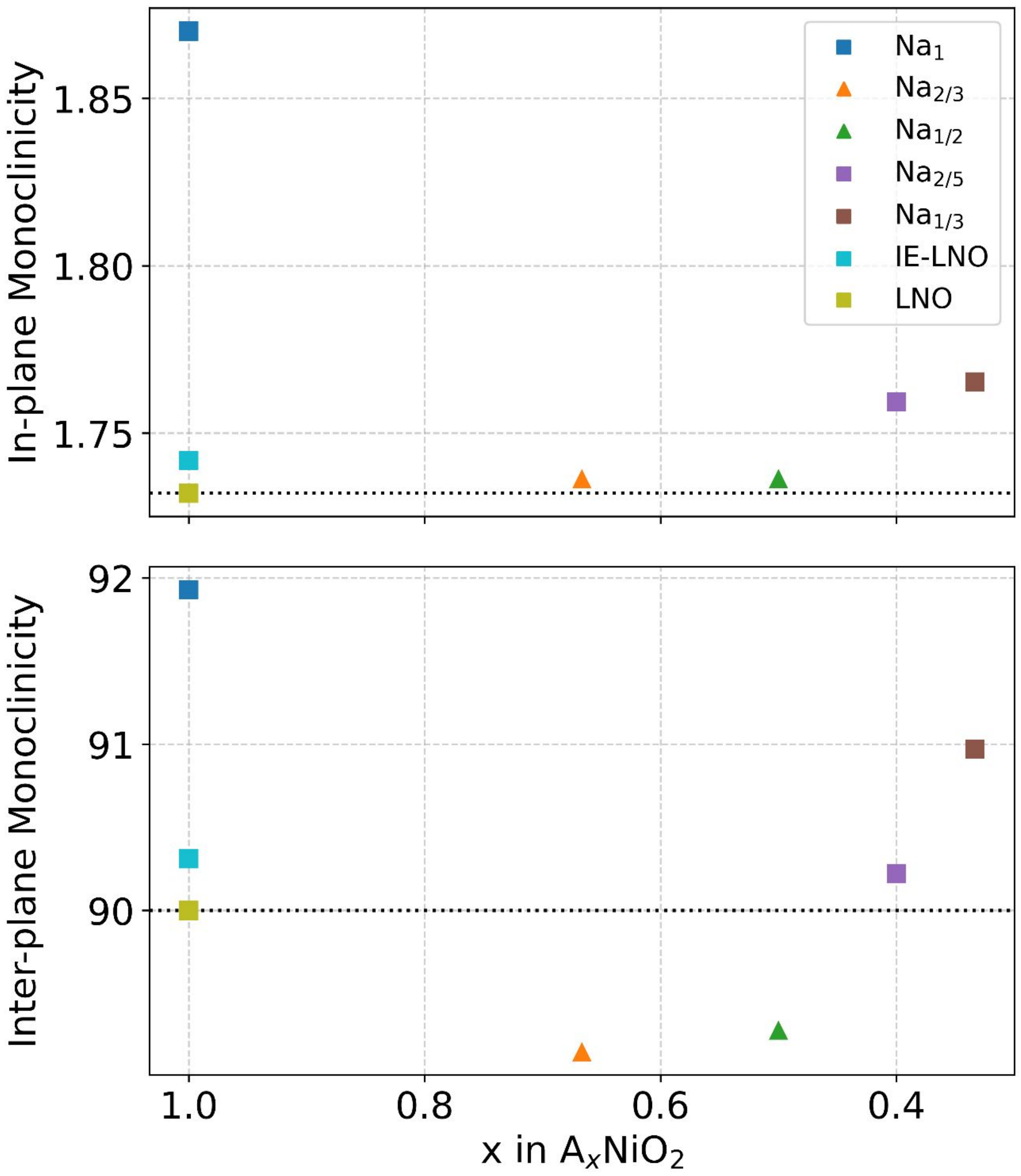

Figure S13: Quantification of in-plane and inter-plane monoclinicity in Na_xNiO_2 and IE-LNO/LNO structures, where deviation from the dotted line ($\sqrt{3}$ *and 90° respectively, representative of no monoclinic distortion, as in defect-contianing $LiNiO_2$) represents greater monoclinicity. Octahedral and prismatic phases are denoted by squared and triangles respectively.*

S-15: Evolution of Octahedral Distortion in First Row A_xTMO_2 Compounds

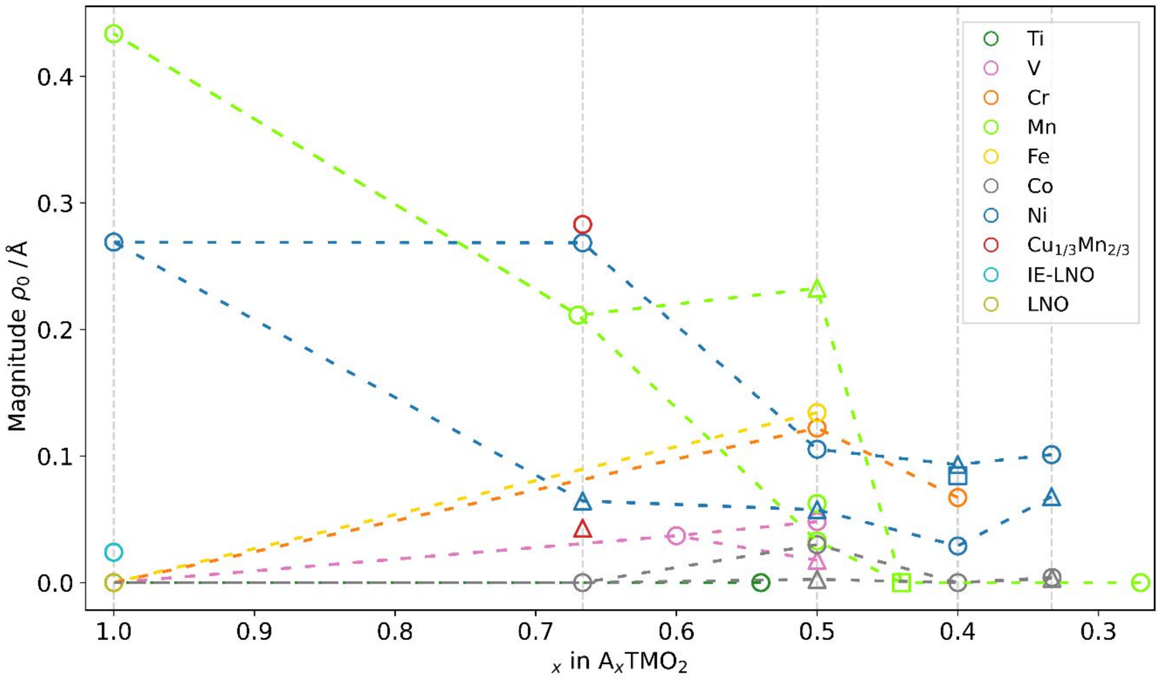

Figure S14: Evolution of magnitude (ρ_0) of the Jahn-Teller distortion with decreasing alkali ion content in first row transition metal oxides. Where multiple sites are present in a structure they are denoted by the shapes of markers in the order circle, triangle, square, from lowest to highest site multiplicity. Structures retrieved from ICSD with collection codes as detailed in Table S5.

Table S5: Collection codes and references for the structures contained in Figure S14.

Composition	ICSD Collection Code or Reference
$NaTiO_2$	85657
$Na_{0.54}TiO_2$	68872
$NaVO_2$	420137
$Na_{0.6}VO_2$	260583
$Na_{0.5}VO_2$	194368
$NaCrO_2$	116507
$Na_{0.5}CrO_2$	Reference 36
$Na_{0.4}CrO_2$	Reference 29
$NaMnO_2$	155330
$NaMn_{0.67}O_2$	117921
$Na_{0.5}MnO_2$	175661
$Na_{0.437}MnO_2$	19656
$Na_{0.272}MnO_2$	19657
$NaFeO_2$	75588
$Na_{0.5}FeO_2$	75589
$NaCoO_2$	96428
$Na_{0.67}CoO_2$	98210
$Na_{0.5}CoO_2$	155492
$Na_{0.4}CoO_2$	138400
$Na_{0.33}CoO_2$	Reference 14
$NaNiO_2$	85317
$Na_{2/3}NiO_2$	Reference 12
$Na_{1/2}NiO_2$	Present work
$Na_{2/5}NaO_2$	Present work
$Na_{1/3}NiO_2$	Present work
$Na_{2/3}Cu_{1/3}Mn_{2/3}O_2$	Reference 44
IE-$LiNiO_2$	Reference 48
$LiNiO_2$	70422
